# Supplementary material for: The landscape and level of alcohol policy enforcement in Tanzania
Source: PLOS Glob Public Health. 2024 Dec 2;4(12):e0003203. doi: 10.1371/journal.pgph.0003203 (PMC11611165; doi:10.1371/journal.pgph.0003203)
Supplement: S1 File — (PDF) [file pgph.0003203.s001.pdf]

World Health Organization
REGIONAL OFFICE FOR Europe

Policy in action

Policy in action

A tool for measuring alcohol policy implementation

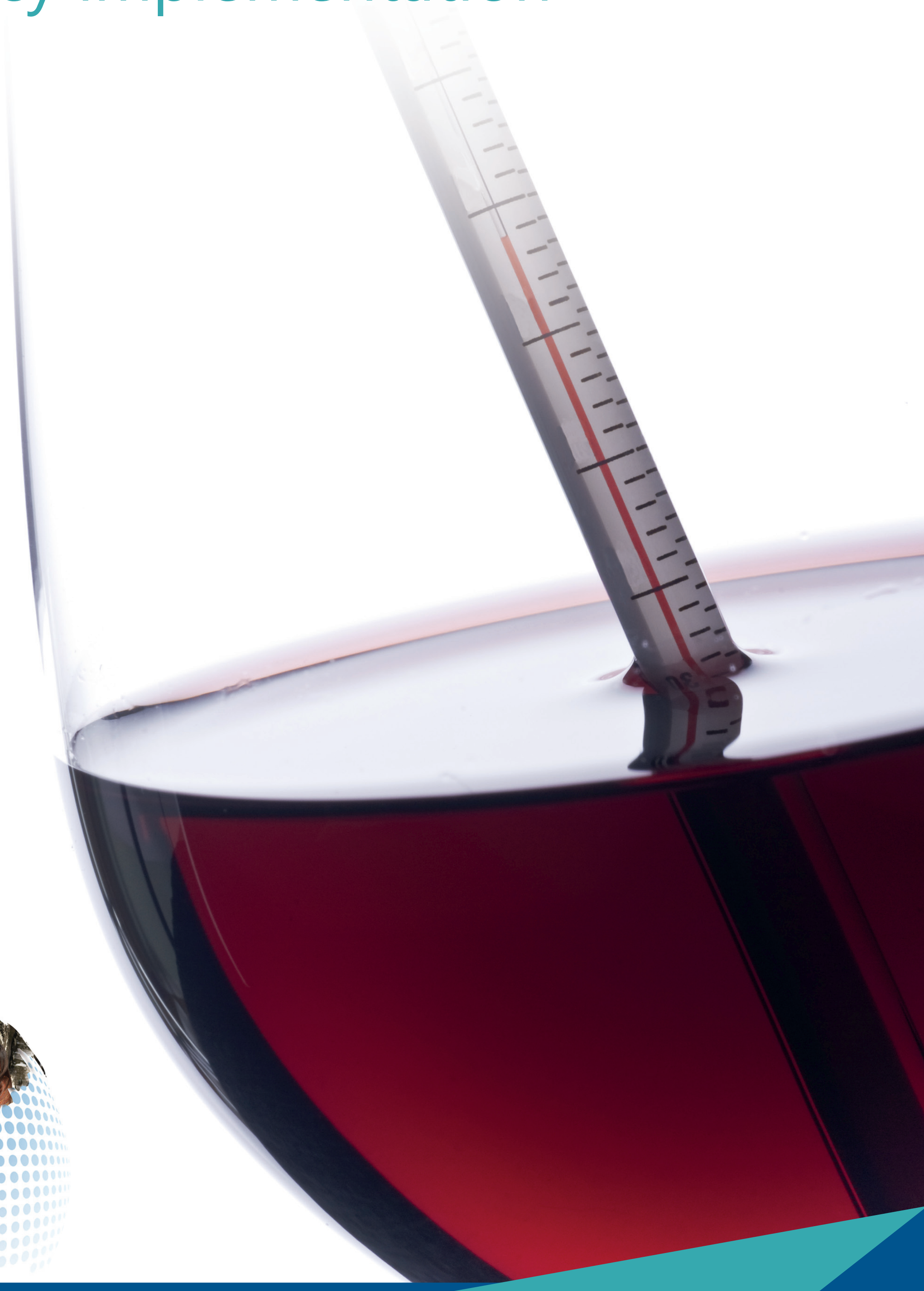

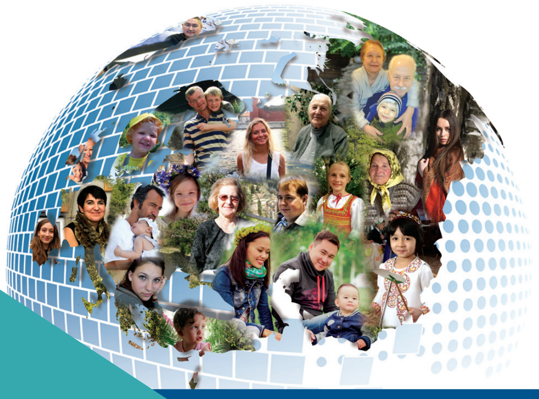

WHO European Office for the Prevention and Control of Noncommunicable Diseases

9 Leontyevsky Pereulok
125009 Moscow, Russian Federation
Email: NCDoffice@who.int

Website:
www.euro.who.int/en/NCDoffice

World Health Organization

REGIONAL OFFICE FOR Europe

POLICY IN ACTION

A tool for measuring alcohol policy implementation

ABSTRACT

Europe has the highest alcohol consumption and alcohol-attributable disease burden in the world. In 2011, all 53 Member States of the WHO European Region endorsed the European action plan to reduce the harmful use of alcohol 2012–2020 (EAPA), which provides a portfolio of evidence-based policy options for mitigating alcohol-associated problems. To assess the extent to which Member States have adopted the recommended policy standards, the WHO Regional Office for Europe has developed 10 composite indicators, one for each action area of the EAPA. This document describes the construction of the EAPA composite indicators and presents an evaluation of the performance of Member States in the European Region in implementing the 10 action areas. The composite indicators measure not only the presence of alcohol policies but also their strictness and comprehensiveness.

Keywords

Alcohol Drinking - adverse effects
Alcohol Drinking - prevention and control
Alcohol-Related Disorders - prevention and control
Alcoholism - prevention and control
Regional Health Planning
Europe

Address requests about publications of the WHO Regional Office for Europe to:

Publications
WHO Regional Office for Europe
UN City, Marmorvej 51
DK-2100 Copenhagen Ø, Denmark

Alternatively, complete an online request form for documentation, health information, or for permission to quote or translate, on the Regional Office web site (http://www.euro.who.int/pubrequest).

Photo: Chiyacat/Shutterstock.com

© World Health Organization 2017

All rights reserved. The Regional Office for Europe of the World Health Organization welcomes requests for permission to reproduce or translate its publications, in part or in full.

The designations employed and the presentation of the material in this publication do not imply the expression of any opinion whatsoever on the part of the World Health Organization concerning the legal status of any country, territory, city or area or of its authorities, or concerning the delimitation of its frontiers or boundaries. Dotted lines on maps represent approximate border lines for which there may not yet be full agreement.

The mention of specific companies or of certain manufacturers' products does not imply that they are endorsed or recommended by the World Health Organization in preference to others of a similar nature that are not mentioned. Errors and omissions excepted, the names of proprietary products are distinguished by initial capital letters.

All reasonable precautions have been taken by the World Health Organization to verify the information contained in this publication. However, the published material is being distributed without warranty of any kind, either express or implied. The responsibility for the interpretation and use of the material lies with the reader. In no event shall the World Health Organization be liable for damages arising from its use. The views expressed by authors, editors, or expert groups do not necessarily represent the decisions or the stated policy of the World Health Organization.

CONTENTS

Acknowledgements . . . v
Abbreviations . . . vi
Foreword . . . vii
Introduction . . . 1
- Alcohol consumption and harm . . . 1
- Global context of alcohol policy . . . 2
- Aims of the composite indicators . . . 3

Methods . . . 4
- Background . . . 4
- Data sources . . . 5
- Construction of scoring scheme . . . 5
- Generation of scores . . . 8
- Scoring scheme rationale . . . 9

Results . . . 12
- Scoring scheme . . . 12
- Regional scores . . . 13

Discussion . . . 18
- Summary of findings . . . 18
- Improvements from previous composite indicators . . . 18
- Policy interactions . . . 19
- Robustness of the EAPA composite indicators . . . 19
- Strengths and limitations of the EAPA composite indicators . . . 20
- Future work . . . 21

Conclusion . . . 22
References . . . 23
Annex 1. List of survey questions used for the EAPA composite indicators arranged by SIs . . . 28
Annex 2. Detailed scoring rubrics for the EAPA composite indicators . . . 41

ACKNOWLEDGEMENTS

This first draft of this report was prepared by Genim Tan, Consultant, WHO Regional Office for Europe. Technical editing was provided by: Julie Brummer, Consultant; Lisa Schölin, Consultant; Lars Møller, Programme Manager; and Gauden Galea, Director, Division of Noncommunicable Diseases and Promoting Health through the Life-Course, WHO Regional Office for Europe.

The project was carried out in association with Thomas Karlsson, Esa Österberg and Mikaela Lindeman of the WHO Collaborating Centre on Alcohol Policy Implementation and Evaluation, Finland.

The project had an expert advisory group with the following members: Thomas Babor, University of Connecticut School of Medicine, Department of Community Medicine & Health Care, United States of America; Bernt Bull, Ministry of Health and Care Services, Department of Public Health, Norway; Vesna-Kerstin Petri, Division for Health Promotion and Prevention of Noncommunicable Diseases, Ministry of Health, Slovenia; Emanuele Scafato, WHO Collaborating Centre for Research and Health Promotion on Alcohol and Alcohol-Related Health Problems, National Observatory on Alcohol, National Health Institute, Italy; Esa Österberg, National Institute for Health and Welfare, Finland; Tatiana Klimenko and Konstantin Vyshinskiy, Federal Medical Research Centre for Psychiatry and Narcology, Ministry of Health, Russian Federation; Vladimir Poznyak, WHO headquarters; Gauden Galea, Lars Møller and Julie Brummer, WHO Regional Office for Europe; and Jürgen Rehm, Pan American Health Organization/WHO Collaborating Centre for Mental Health and Addiction, Canada; Institute for Mental Health Policy Research and Campbell Family Mental Health Research Institute, Centre for Addiction and Mental Health, Canada; Institute of Medical Science, Department of Psychiatry and Dalla Lana School of Public Health, University of Toronto, Canada; Institute for Clinical Psychology and Psychotherapy, Technical University, Dresden, Germany.

Imke Seifert, Intern, and Renée Bouhuijs, Consultant, WHO Regional Office for Europe, assisted with data collection and verification.

The document benefited from input from the following peer reviewers: Charlie Foster, Associate Professor of Physical Activity and Population Health and Deputy Director, British Heart Foundation Centre on Population Approaches for NCD Prevention, Nuffield Department of Population Health, University of Oxford, United Kingdom; and Emma Plugge, Senior Clinical Research Fellow, Centre for Tropical Medicine and Global Health, Nuffield Department of Clinical Medicine, University of Oxford, United Kingdom.

The project was carried out by the WHO Regional Office for Europe in the context of the Project on the Prevention and Control of NCDs, financed by the Ministry of Health of the Russian Federation.

ABBREVIATIONS

AMPHORA	Alcohol Measures for Public Health Research Alliance
APC	adult per capita consumption
APS	Alcohol Policy Scale
ATLAS-SU	ATLAS on Substance Use
BAC	blood alcohol concentration
EAPA	European action plan to reduce the harmful use of alcohol 2012–2020
EISAH	European Information System on Alcohol and Health
EU	European Union
GDP	gross domestic product
ICD-10	International Classification of Diseases, 10th revision
OECD	Organisation for Economic Co-operation and Development
PDS	pattern of drinking score
PPP	purchasing power parity
RSUD	System on Resources for the Prevention and Treatment of Substance Use Disorders
SI	summary indicator
TEASE	Toolkit for Evaluating Alcohol policy Stringency and Enforcement

FOREWORD

In September 2011, the European action plan to reduce the harmful use of alcohol 2012–2020 (EAPA) was endorsed by all 53 Member States in the WHO European Region. The action plan lays out a range of evidence-based policy options aimed at restricting the supply of, and reducing the demand for, alcohol. It is the latest in a series of policy instruments developed to guide Member States in the European Region, a process which began approximately 20 years ago with the endorsement of the first alcohol action plan.

By resolution EUR/RC61/R4 endorsing the EAPA, the WHO Regional Committee for Europe recommended that Member States in the European Region use the action plan to formulate or, if appropriate, reformulate national alcohol policies and action plans, and requested that the Regional Director monitor the progress, impact and implementation of the European action plan. At the request of Member States, the Regional Office produced a list of indicators which could be used as a tool to support them in the implementation, evaluation and monitoring of individual national alcohol policies.

This report describes the construction of 10 novel composite indicators, which provide a further resource for evaluating the extent to which the policy measures of the action plan have been implemented by Member States. The composite indicators are composed of 34 summary indicators and reflect the 10 action areas of the EAPA. They measure whether a Member State has implemented a policy measure and take into account the level of empirical support for the measure's effectiveness as well as the level of strictness and comprehensiveness of each action. As such, the composite indicators allow monitoring to go beyond solely tracking whether a Member State has a national alcohol policy to a more fine-grained approach of evaluating the individual components.

The need to promote evidence-based alcohol policies in the Region is made even more apparent by data presented in the Global status report on alcohol and health 2014, *which show that the Region continues to lead all WHO regions in alcohol per capita consumption, prevalence of heavy episodic drinking among adults and adolescents and proportion of alcohol-attributable deaths. Given the harm that alcohol can do to individuals and societies, it is time to seek out more refined methods of evaluating national policies to ensure that they reflect the current evidence base. The EAPA composite indicators provide such as a tool, as they convey at a glance the extent to which Member States have adopted the recommended best practices outlined in the action plan and can also be used to monitor trends over time and compare policy options.*

It is our hope that the scoring can be updated regularly by the WHO secretariat, using data from the European Information System on Alcohol and Health.

Gauden Galea
Director, Division of Noncommunicable Diseases and Promoting Health through the Life-Course
WHO Regional Office for Europe

INTRODUCTION

Alcohol consumption and harm

The practice of consuming alcohol transcends temporal and geographical boundaries but its symbolism differs from culture to culture. Alcohol may be associated with celebration and revelry, ritual and religion, individuality or conformity, or simply a quotidian component of the mealtime routine *(1)*. However, beneath these oft-romanticized layers of meaning lies a sobering fact: alcohol is detrimental to health. It is a teratogen, neurotoxin, intoxicant, carcinogen and immunosuppressant *(2)*. Alcohol use was the fifth leading risk factor for the global disease burden in 2010 *(3)* and it is responsible for an estimated 3.3 million deaths every year and 5.1% of disability-adjusted life-years worldwide *(4)*. It was the most important risk factor among people aged 15–49 years *(3)*.

The negative health consequences of alcohol consumption are manifold. More than 30 categories in the *International classification of diseases and related health problems, 10th revision* (ICD-10) consist of conditions wholly attributable to alcohol *(5,6)*, including alcohol use disorders, alcoholic psychoses and alcoholic gastritis *(1)*. In addition, alcohol is a component cause for more than 200 ICD-10 three-digit codes covering categories of disease such as cancer, cardiovascular disease and metabolic dysfunction *(7)*. Although there is some evidence for the protective effects of light sporadic drinking on coronary heart disease, ischaemic stroke and diabetes *(8,9)*, the adverse effects of alcohol still preponderate *(10)*. Besides chronic diseases that manifest themselves after years of cumulative drinking, significant morbidity and mortality also result from acute injury.

The brunt of the harm related to alcohol is not borne by drinkers alone. Many undesirable consequences spill over into the realm of the family and wider community. Societal harms associated with drinking include the deterioration of personal and working relationships, criminal behaviour (such as vandalism and violence), productivity losses and substantial health care costs *(10,11)*. Together, the alcohol-attributable disease burden and costs to society translate into approximately 1.3% of the gross domestic product (GDP) in European Union (EU) countries *(12)*. Importantly, alcohol also contributes to inequities within and between countries. There is strong evidence that alcohol use and harm vary along the socioeconomic gradient *(13,14)*, with lower socioeconomic groups experiencing greater harm despite lower levels of consumption, known as the alcohol harm paradox. This is particularly true among the younger age groups and among men *(15)*.

Alcoholic beverages are available in almost all parts of the world, but the importance of alcohol as a risk factor depends largely on the way it is consumed. The two indicators particularly relevant to health are adult per capita consumption (APC) and pattern of drinking score (PDS) (Table 1). For populations with equivalent APC, a higher PDS is associated with less favourable health outcomes *(16)*. The worldwide APC was 6.4 litres in 2014. However, the global average conceals significant variations in consumption between geographical regions. The APC in the WHO European Region was 10.7 litres; at the other end of the spectrum, an APC of 0.6 litres was reported for the Eastern Mediterranean Region *(17)* where, based on 2010 data, 89.8% of the adult population are lifetime abstainers *(4)*. In the WHO European Region, the lowest PDS are found in only a handful of countries in southern and western Europe, while the riskiest drinking patterns are prevalent in the Russian Federation and Ukraine *(4)*. Heavy drinking occasions are particularly harmful to health and are important contributors to injury and cardiovascular mortality. Since 1990, the alcohol-attributable mortality burden in the European Region has increased, largely owing to trends in the eastern part of the Region, which saw a 22% increase *(17)*.

Table 1. Pathways of alcohol-related harm

Indicator	Definition
APC	Average volume in litres of pure alcohol consumed by people aged 15 years and older.
PDS	A measure of how hazardous the drinking behaviour is in a population on a scale from 1 (least risky) to 5 (most risky). It is calculated on the basis of: (i) the usual quantity of alcohol consumed per drinking occasion; (ii) the prevalence and frequency of festive drinking; (iii) the proportion of drinking events when drinkers become intoxicated; (iv) the proportion of drinkers who drink daily or nearly every day; (v) the prevalence of drinking with meals; and (vi) the prevalence of drinking in public places *(4)*.

Global context of alcohol policy

Momentum in international alcohol policy has gathered pace slowly but surely. For many years, the European Region has had the highest level of alcohol consumption, which has led the Regional Office to take a leading role in joint political action to tackle alcohol use and harm. Since the launch of the pioneering European alcohol action plan in 1992, alcohol has continued to feature regularly in the activities of the Regional Office as well as on the agendas of other regional offices and at the World Health Assembly (Table 2). These culminated, in May 2010, in the adoption of resolution WHA63.13 that endorses the global strategy to reduce the harmful use of alcohol *(18)*. Through a broad consultation process involving multiple stakeholders, all 193 WHO Member States arrived at this historical consensus on ways to ameliorate alcohol-related harm *(19)*. The aims of the global strategy are to increase the commitment by governments, strengthen the knowledge base, enhance the capacity of Member States, foster partnerships and coordination, and improve monitoring and surveillance systems in order to curb the harmful use of alcohol *(18)*. The strategy also includes a recommended portfolio of evidence-based interventions grouped into 10 action areas (Table 3). The Regional Office subsequently drew up the *European action plan to reduce the harmful use of alcohol 2012–2020* (EAPA), which was adopted by all 53 Member States in the European Region in September 2011 *(2)*. The EAPA is aligned seamlessly with the WHO global strategy and contains a mixture of policy options aimed at restricting the supply of and reducing the demand for alcohol. These include restrictions on advertising, excise taxes, a minimum purchase age, brief interventions in health care and workplace treatment programmes.

Table 2. History of WHO's activity in international alcohol policy, 1992–2011

Year	WHO body	Action
1992	WHO Regional Committee for Europe	European alcohol action plan 1992–1999 (WHO Regional Committee for Europe resolution EUR/RC42/R8)
1995	WHO Regional Office for Europe	European charter on alcohol (adopted at the European Conference on Health, Society and Alcohol, 1995)
1999	WHO Regional Committee for Europe	European alcohol action plan 2000–2005 (WHO Regional Committee for Europe resolution EUR/RC49/R8)
2001	WHO Regional Committee for Europe	Declaration on young people and alcohol (WHO Regional Committee for Europe resolution EUR/RC51/R4)
2005	WHO headquarters	Public health problems caused by harmful use of alcohol (World Health Assembly resolution WHA58.26)
2005	WHO Regional Committee for Europe	Framework for alcohol policy in the WHO European Region (WHO Regional Committee for Europe resolution EUR/RC55/R1)
2006	WHO Regional Committee for South-East Asia	Alcohol consumption control – Policy options in the South-East Asia region (WHO Regional Committee for South-East Asia resolution SEA/RC59/15)
2006	WHO Regional Committee for the Western Pacific	Regional strategy to reduce alcohol-related harm (WHO Regional Committee for the Western Pacific resolution WPR/RC57.R5)
2006	WHO Regional Committee for the Eastern Mediterranean	Public health problems of alcohol consumption in the Eastern Mediterranean Region (WHO Regional Committee for the Eastern Mediterranean resolution EM/RC53/R.5)
2007	WHO headquarters	WHO Expert Committee on Problems Related to Alcohol Consumption (WHO Technical Report Series, No. 944, 2007)
2008	WHO headquarters	Strategies to reduce the harmful use of alcohol (World Health Assembly resolution WHA61.13)
2010	WHO headquarters	Global strategy to reduce the harmful use of alcohol (World Health Assembly resolution WHA63.13)
2010	WHO Regional Committee for Africa	Reduction of the harmful use of alcohol: a strategy for the WHO African Region (WHO Regional Committee for Africa resolution AFR/RC60/R2)
2011	WHO Regional Office for the Americas	Plan of action to reduce the harmful use of alcohol (WHO Regional Committee for the Americas resolution CD51.R14)
2011	WHO Regional Committee for Europe	European action plan to reduce the harmful use of alcohol 2012–2020 (WHO Regional Committee for Europe resolution EUR/RC61/R4)

Sources: WHO *(2,4,18)*; Babor *(11)*; Rekve *(20)*.

Table 3. The global strategy to reduce the harmful use of alcohol: areas for policy options and interventions

Target areas	Options for policies and interventions
Leadership, awareness and commitment	Expressing political commitment through adequately funded, comprehensive and intersectoral national policies that are evidence-based and tailored to local circumstances
Health services' response	Providing preventive services and treatment to individuals and families at risk of, or affected by, alcohol-use disorders and associated conditions
Community and workplace action	Harnessing the local knowledge and expertise of communities to change collective behaviour
Drink–driving policies and countermeasures	Introducing measures to deter people from driving under the influence of alcohol; creating a safer driving environment to minimize the likelihood and severity of alcohol-influenced road traffic accidents
Availability of alcohol	Preventing easy access to alcohol for vulnerable and high-risk groups; reducing the social availability of alcohol so as to change social and cultural norms that promote the harmful use of alcohol
Marketing of alcoholic beverages	Protecting young people by regulating both the content of alcohol marketing and the amount of exposure to that marketing
Pricing policies	Increasing the prices of alcoholic beverages to reduce underage drinking, to halt progression towards drinking large volumes of alcohol and/or episodes of heavy drinking, and to influence consumers' preferences
Reduction of the negative consequences of drinking and alcohol intoxication	Reducing the harm from alcohol intoxication by managing the drinking environment and informing consumers
Reduction of the public health impact of illicit alcohol and informally produced alcohol	Reducing the negative consequences of informal or illicit alcohol through good market knowledge, an appropriate legislative framework and active enforcement of measures
Monitoring and surveillance	Developing surveillance systems to monitor the magnitude of and trends in alcohol-related harms, to strengthen advocacy, to formulate policies and to assess the impact of interventions

Source: WHO *(18)*.

Aims of the composite indicators

In spite of the policy resources made available by the Regional Office, countries in Europe continue to be affected by alarming levels of alcohol-attributable harm. In the European Region, alcohol has a causal impact in approximately 15% of all causes of death *(17)*. This suggests that there is a gap between what is known and what is practised. If that is the case, how can the extent to which governments have adopted the recommended best practices reflected in the European action plan be determined? One way of measuring multidimensional phenomena (such as countries' performance as regards alcohol policy) is by compiling individual indicators into a composite indicator on the basis of an underlying model *(21)*. Such aggregated indices are found in numerous research and policy fields and are typically used to make comparisons between organizations, institutions or countries *(22)*. Well-known examples include the Human Development Index *(23)*, the Global Competitiveness Index *(24)*, the Corruption Perceptions Index *(25)*, the overall health system attainment *(26,27)* and the Better Life Index *(28)*. The appeal of composite indicators lies in their ability to convey, at a glance, a large amount of information that is relevant to decision-making and priority-setting. This report describes the construction of 10 novel composite indicators that quantify the completeness of national alcohol strategies and plans (that is, the number of policies that are present and the degree to which each policy meets certain prescribed standards). The extent to which actions in the policy areas of the EAPA have been implemented by Member States in the Region is also described in this report, as well as the strengths and limitations of the composite indicators.

METHODS

Background

There is no gold standard methodology for constructing composite indicators. It depends on the "craftsmanship of the modeller" and is assessed on a fitness for purpose basis. The quality of a composite indicator boils down to the quality of the conceptual framework and data sources used *(21)*.

One important consideration is the weight that each component of the indicator should be assigned. In other words, should all components matter equally, or should some components be given more weight? A further option is to leave the question open, to be answered by the individual user. The Organisation for Economic Co-operation and Development (OECD) used this last approach in creating the Better Life Index. This Index makes use of an interactive platform that allows each user to vary the weights of the 11 dimensions, including education, health and work-life balance, and to observe the effects on country rankings of well-being *(28)*.

The fluid approach employed by the Better Life Index makes it clear that there is no single way to assign weights. Traditionally, however, developers of composite indices have used a more static method for assigning weights. The Human Development Index, which is published by the United Nations Development Programme, uses an equal weighting approach. The Human Development Index includes the following three dimensions: long and healthy life (also referred to as health, as measured by the indicator life expectancy at birth), knowledge (also referred to as education, as measured by the arithmetic mean of the indicators mean years of schooling and expected years of schooling) and a decent standard of living (also referred to as income, as measured by the indicator gross national income per capita (in purchasing power parity (PPP) international dollars)). The Human Development Index for a Member State is calculated based on the geometric mean of the three dimension indices, where each dimension has equal weight *(23)*.

Another well-known example is the overall health system attainment composite measure, published by WHO in 2000, which consists of five components: health, health inequality, responsiveness, responsiveness inequality and fairness of financial contribution. In order to determine the weights assigned for each component, an internet survey was conducted among WHO staff members (from headquarters, regional and country offices) and visitors to the WHO website. These participants were assumed to have specialized knowledge of the topic based either on their employment at WHO or their interest in the WHO website *(26,27)*.

In the area of alcohol policy, a recent project to assess the effect of the United States of America's alcohol control policy environment on drinking behaviour evaluated both the equal and differential weighting approaches *(29,30)*. The Alcohol Policy Scale (APS) is a composite measure that was created to assess the relationship between alcohol policy measures, which vary by state, and harmful drinking behaviours. To develop the APS, a panel of 10 experts was tasked with putting forward suggestions for effective policies to be included in the composite measure and with assigning ratings of efficacy (that is, effectiveness of the policy in reducing the harmful use of alcohol) and implementation (the strictness of the policy). The methodology involved an initial individual web-based survey of the experts, a face-to-face panel discussion, and a follow-up individual expert survey to finalize the efficacy and implementation ratings. The researchers evaluated several methods for constructing the APS, including those that involved equal weighting (that is, summing the existing policies in each state, with one point given per policy) and methods that accounted for efficacy and implementation ratings. APS scores generated by all methods were significantly associated with drinking outcomes; methods that took into account efficacy and implementation ratings resulted in a better fit *(29,30)*.

Other relevant efforts to quantitatively compare the overall policy stance of national governments on alcohol have assigned differential weights based on expert opinion and reviews of the evidence base (see the Alcohol Measures for Public Health Research Alliance (AMPHORA) project scale *(31)*, the Alcohol Policy Index *(32)* and the Toolkit for Evaluating Alcohol policy Stringency and Enforcement-16 (TEASE-16) *(33)*). The Alcohol Policy Index and TEASE-16 projects also included evaluations of different weighting structures as part of the sensitivity analysis.

Overview of methods used to construct the EAPA composite indicators

For the current project, the EAPA was chosen as a scaffold for a selection of policy variables to be subsumed into the composite indicators. The EAPA contains a broad spectrum of policy instruments that are consistent with current evidence-based recommendations. This improves the validity of the content of the composite indicators by ensuring that all important facets of a national alcohol policy are accounted for *(34)*. Furthermore, the Regional Office has established procedures for collecting policy information on indicators corresponding to each action area, thereby minimizing problems associated with missing or inconsistent data. Lastly, because the EAPA has been endorsed by all 53 Member States in the European Region, composite indicators that mirror the action plan are more likely to gain traction among public health leaders and policy-makers.

The EAPA composite indicators were developed and evaluated in two phases. The aim of the first phase was to construct a scoring scheme by aggregating, scaling and weighting selected policy indicators. This phase was carried out via a face-to-face meeting of the project's expert advisory group and subsequent e-mail consultations. In the second phase, relevant policy data for the Member States were collected and coded, and composite indicator scores were computed for each country for which there were sufficient data. The project methodology was informed by technical handbooks on the development of composite indicators *(21,35)* as well as previous work done in the area of alcohol control indices *(31,32)*. Details of each phase will be explained in the subsequent sections.

Data sources

The main data sources for this project were the European Information System on Alcohol and Health (EISAH) and the European Regional Information System on Resources for the Prevention and Treatment of Substance Use Disorders (RSUD). These databases for the WHO European Region contain alcohol-related indicators at the country level.

WHO's principal tool for amassing information from all Member States on alcohol control policies, alcohol consumption, alcohol-related health consequences as well as national monitoring and surveillance systems is the global survey on alcohol and health. In the European Region, the Regional Office and the European Commission jointly administer a modified version of the global survey instrument. WHO's main tool for assessing and monitoring health system resources worldwide related to substance use disorders is the ATLAS on Substance Use (ATLAS-SU) questionnaire. These WHO surveys take the form of a self-completion questionnaire. Designated national experts are asked to fill out the questionnaire in consultation with other experts from their respective countries. Survey data are then uploaded to regional and global alcohol databases maintained by WHO, including EISAH and RSUD.

Data for this project are largely based on the global survey on alcohol and health conducted in 2012 and the substance use ATLAS-SU questionnaire conducted in 2014. Responses from the WHO global questionnaire on progress in alcohol policy, administered in 2015, were used to update the indicators also included in this questionnaire, and national experts nominated as contact persons for WHO were contacted by e-mail in June 2016 to confirm or update existing data. The most recent available data were used to generate the composite indicators.

Estimates of gross national income at PPP for 2015 were obtained from the World Bank *(36)*.[1,2]

Construction of scoring scheme

The purpose of the scoring scheme was to put in place a logical and consistent process by which, for each country, a large volume of policy information could be condensed into a score for each of the 10 action areas of the EAPA. Important considerations during this phase were that:

- countries with stronger policies should receive more credit than those with weaker policies, but it should be possible in theory for all Member States in the European Region to attain the maximum score;
- all 10 EAPA action areas should be represented and, within an action area, policy options that are more actively promulgated by WHO should be accorded higher priority; and
- the scoring scheme should be grounded in scientific evidence and reflect current best practices.

[1] As World Bank data were unavailable for Andorra, an estimate of the 2014 gross national income per capita (at 2011 PPP international dollars) was taken from the Human Development Report *(37)*.

[2] The most recent World Bank estimate for Malta is from 2013.

At a meeting held at the Regional Office in April 2015, an expert advisory group selected a subset of survey questions from the WHO questionnaires that would be most illuminating in the context of policy benchmarking and evaluating the implementation of the EAPA. The chosen questions were then grouped into thematic clusters. Because policy variables within each cluster were conceptually related, they could be subsumed under a common summary indicator (SI). In this sense, each SI measures a particular aspect of alcohol control and serves as a building block for the composite indicator for each EAPA action area. Examples of SIs include restrictions on alcohol availability by time, community-based interventions to reduce alcohol-related harm and legally binding restrictions on product placement. It was necessary to reformulate and recode existing variables in the creation of certain SIs. This will be explained in a subsequent section. The final 34 SIs were categorized in the 10 EAPA action areas. The complete list of survey questions used in this project is presented in Annex 1.

Since it was desirable for information to be aggregated with minimal loss of precision, scales were introduced to distinguish different degrees of success within each SI. Depending on the nature of the topic, the scale might reflect a gradient in stringency (such as legal age limits) or comprehensiveness (such as the scope of the monitoring system).

A nested banding approach was employed for the indicators pertaining to marketing (indicators 6.1 to 6.4) and affordability (indicator 7.2). With regard to the former, points are awarded for multiple items (such as various advertising platforms) based on the level of restriction applied to different types of beverage (details of the scoring scheme are in Annex 2). The sum of points across the items corresponds to a band, which in turn determines the final score for the indicator. This methodology follows that of Esser & Jernigan *(38)*. An example is shown in Table 4. In the case of affordability, the band is ascertained according to the price indices of different types of beverage. The price index is a modification of the affordability measure first introduced by Brand et al. *(31)* and is defined as follows:

Price index

$$= 10\,000 \text{ X } \frac{\text{Price (calculated based on standard containers of 50 cl beer, 75 cl wine and 70 cl spirits) (€)}}{\text{Gross national income at PPP per capita (current international \$)}}$$

Table 4. Example of a score for legally binding restrictions on product placement (indicator 6.2) following the nested banding approach[a]

Item	Beverage type	Restriction	Points (level of restriction)
National television	Beer	Ban	3
	Wine	Partial statutory	2
	Spirits	Voluntary	1
Cable television	Beer	None	0
	Wine	Ban	3
	Spirits	Ban	3
Films	Beer	Ban	3
	Wine	Ban	3
	Spirits	Ban	3
		Total points	*21*
		Band	*4*
		Final score for indicator	*12*

[a] *See* Annex 2, Rubric 6

As well as the nuances within each policy topic, the differential effectiveness between policies in an action area was also factored into the construction of the scoring scheme. Rather than taking all potential interventions to be on an equal footing, each SI was weighted according to the strength of the underlying evidence.

The product of the raw score and the multiplier level produces a weighted score for each SI. The total score for the action area is a linear summation of all the SIs.

Members of the expert advisory group provided the first round of input on the scales and weights for each SI via e-mail consultations in June 2015. The Regional Office and the WHO Collaborating Centre on Alcohol Policy Implementation and Evaluation developed the scoring rubric based on the experts' feedback and the publication *Alcohol: no ordinary commodity (11)*. Numerous policy measures are evaluated in the book and given a rating of 0–3 on the three dimensions of effectiveness, breadth of research support and extent of cross-national testing. These quantitative ratings were transposed into five multiplier levels for the current project (Table 5). Other publications providing a synthesis of available evidence were also used to guide the allocation of multiplier levels *(10,39)*. The scoring rubric was submitted to the expert advisory group for final review in October 2015.

Table 5. Description of a tool used for weighting SIs

Multiplier level	Description	Ratings by Babor et al.[a]
5x	High level of effectiveness demonstrated consistently across different populations OR fundamental public health infrastructure needed to initiate and sustain an effective response	• Effectiveness: 3<br>• Breadth of research support/ cross-national testing: 2 or 3
4x	High level of effectiveness demonstrated in a limited number of studies and populations OR moderate effectiveness demonstrated consistently across different populations	• Effectiveness: 3<br>• Breadth of research support/ cross-national testing: 1 or 2<br>OR<br>• Effectiveness: 2<br>• Breadth of research support/ cross-national testing: 2 or 3
3x	Moderate effectiveness demonstrated in a limited number of studies and populations	• Effectiveness: 2<br>• Breadth of research support/ cross-national testing: 1 or 2
2x	Limited effectiveness OR insufficient evidence to conclude degree of effectiveness	• Effectiveness: 1<br>OR<br>• Effectiveness: unknown
1x	Not shown on its own to be effective but may be valuable as part of a package of policy measures	• Effectiveness: 0

[a] Babor et al. *(11)*.

In sum, the composite indicators were premised on a conceptual framework (the EAPA) and a systematic evidence-based approach was used to define the constituent indicators and their attached weights. Alternative statistical techniques for constructing composite indicators were initially considered. For example, principal component analysis and factor analysis may be employed to "[group] together individual indicators which are collinear to form a composite indicator that captures as much as possible of the information common to individual indicators" *(21)*. These methods are used for reasons of parsimony and to prevent the double counting of overlapping variables. It was decided, however, that a statistical approach was unsuitable given the intended application of the EAPA composite indicators as a tool for political advocacy. It must be clear that statistical correlations "do not necessarily correspond to the real-world links and underlying relationships between the indicators and the phenomena being measured" *(35)*. All meaningful items in the EAPA, regardless of their statistical contribution to the overall variance, ought to be retained in the composite indicators as an indication of their practical importance. Moreover, a composite indicator that is solidly embedded in theory and accompanied by a transparent scoring system is more likely to resonate with policy-makers than an abstract statistical construct. The steps involved in constructing the scoring scheme are illustrated in Fig. 1.

Fig. 1. Illustration of steps taken to construct the scoring scheme, using indicator 1.1 as an example

Step 1: identify survey questions

Is there a written national policy on alcohol specific to your country?

Is the written national policy on alcohol multisectoral?

Is there a national action plan for the implementation of the written national policy on alcohol?

Is a written national policy on alcohol currently being developed or is an adopted one being revised?

↓

Step 2: group into policy topics

↓

Step 3: reformulate variables and establish scales

↓

Step 4: assign multiplier level

**Status**	**Points**		
Written national policy on alcohol	☐ Adopted (2)	☐ In development (1)	☐ No (0)
Written national policy on alcohol is multisectoral	☐ Yes (1)	☐ N/A (0)	☐ No (0)
Written national policy on alcohol policy is accompanied by a national action plan for implementation	☐ Yes (1)	☐ N/A (0)	☐ No (0)
Multiplier level	3x		

Generation of scores

Responses of Member States in the European Region to the relevant survey questions were first retrieved from the datasets compiled by WHO. As described in the section on data sources, national experts nominated as contact persons for WHO were given the opportunity to update responses in June 2016. The most recent available data were used.

Missing values were replaced with zero points. If a substantial portion (>20%) of the data was missing in an action area, the composite indicator was not calculated for that Member State.

Policy variables from the datasets were recoded manually to achieve compatibility with the scoring scheme. To illustrate, the original EISAH dataset for restrictions on alcohol availability by time contains 12 binary variables for the different permutations of on-premise service or off-premise sale,[3] restriction by hours or days of operation and beverage type. These variables were merged into a single SI (indicator 5.3) and recoded following the ordered categories (0, 1, 2, 3, 4) delineated in the scoring scheme (Table 6).

[3] On-premise service refers to alcoholic beverages sold for consumption within the setting of a bar, cafe or restaurant, while off-premise sale refers to alcoholic beverages sold by shops (such as supermarkets and petrol kiosks) for consumption elsewhere.

Table 6. Three possible combinations of values for alcohol availability by time[a]

Variables	Country A	Country B	Country C
On-premise/hours/beer	Yes	Yes	No
On-premise/hours/wine	Yes	Yes	No
On-premise/hours/spirits	Yes	Yes	No
On-premise/days/beer	No	No	No
On-premise/days/wine	No	No	Yes
On-premise/days/spirits	No	No	Yes
Off-premise/hours/beer	Yes	No	No
Off-premise/hours/wine	Yes	No	No
Off-premise/hours/spirits	Yes	No	No
Off-premise/days/beer	No	No	No
Off-premise/days/wine	No	No	Yes
Off-premise/days/spirits	No	No	Yes
Raw score	*4*	*3*	*2*
Final weighted score for indicator 5.3	*12*	*9*	*6*

[a] *See* Annex 2 for details of the scoring scheme.

Table 7 indicates the number of composite indicator scores generated for each action area; that is, the number of Member States for which at least 80% of the data were available.

Table 7. Number of Member States participating in each action area

Action area	Number of Member States participating
Leadership, awareness and commitment	47
Health services' response	34
Community and workplace action	47
Drink–driving policies and countermeasures	53
Availability of alcohol	53
Marketing of alcoholic beverages	53
Pricing policies	45
Reduction of the negative consequences of drinking and alcohol intoxication	52
Reduction of the public health impact of illicit alcohol and informally produced alcohol	53
Monitoring and surveillance	52

Scoring scheme rationale

Because it is impracticable to expound in this report the intricacies of each action area, a summary of the underlying research and scoring assumptions for selected indicators is shown in Table 8. In this section, the principles and assumptions behind two of the best buy interventions recommended by WHO to reduce harmful drinking and thereby the burden of noncommunicable diseases – pricing and marketing – will be explained since they involve more complex data manipulation in the computation of scores.

Table 8. Overview of research evidence and scoring principles for selected indicators

Indicator	Policy rationale and scoring assumptions
1.4 Awareness activities	Most public education campaigns do not lead to sustained changes in alcohol-related behaviour *(11)* apart from those targeting drink–driving *(40)*. Awareness activities are nonetheless important for imparting information and garnering support for alcohol policies *(41)*.<br>*Assumption: awareness activities on more topics lead to a better informed population.*
2.1 Screening and brief interventions for harmful and hazardous alcohol use	Brief interventions in primary care settings produce clinically significant reductions in drinking among non-dependent high risk drinkers *(42)*.<br>*Assumption: insufficient motivation and confidence among practitioners have been cited as important barriers to scaling up brief interventions. It is assumed that this can be ameliorated with adequate training and standardization of guidelines (43).*
3.2 Workplace-based alcohol problem prevention and counselling	There is limited evidence that workplace programmes, such as peer support, can reduce the harm from alcohol *(41)*.
3.3 Community-based interventions to reduce alcohol-related harm	Multicomponent community programmes can be useful for mobilizing communities, changing collective behaviour and increasing the enforcement of alcohol policies *(41)*.
4.1 Maximum legal blood alcohol concentration (BAC) limit when driving a vehicle	The risk of a road traffic accident increases exponentially with BAC and is significantly elevated above a BAC of 0.5 g/litre *(44)*. Lower legal BAC limits are preferred because impairment occurs even at very low BAC levels *(45)*.
4.2 Enforcement using sobriety checkpoints<br>4.3 Enforcement using random breath-testing	Strategies that increase drivers' perceived risk of arrest are effective in deterring drink–driving *(11)*.
5.1 Lowest age limit for on-premise alcohol service and off-premise alcohol sale	A higher minimum legal drinking age is associated with lower alcohol consumption and fewer road traffic accidents *(46)*.<br>*Assumption: on-premise alcohol service and off-premise alcohol sale are assumed to be substitutes. Different beverage types are assumed to be substitutes.*
5.2 Control of retail sales	State-owned monopolies are the most effective structural arrangement for the regulation of alcohol availability. The next best alternative is a licensing system that dictates which vendors may sell alcohol and the exact conditions of sale *(47)*.
5.3 Restrictions on alcohol availability by time	Extending trading hours by a mere one to two hours results in a significantly higher incidence of assaults, motor vehicle accidents and fall-related injuries *(48,49)*.<br>*Assumption: on-premise alcohol service and off-premise alcohol sale are assumed to be substitutes. Different beverage types are assumed to be substitutes.*
5.4 Restrictions on alcohol availability by place	The greater the number of establishments that sell alcohol, the easier it is to obtain alcohol. There is consistent evidence of a positive relationship between the density of outlets and alcohol-associated problems *(11)*.<br>*Assumption: on-premise alcohol service and off-premise alcohol sale are assumed to be substitutes. Different beverage types are assumed to be substitutes.*
5.6 Alcohol-free public environments	Drinking bans in public places potentially reduce drinking and social access to alcohol among young people *(11)*.
7.3 Other price measures	Price increases on cheap alcohol have the most dramatic impact on consumption *(50)*. Discounting results in heavier drinking on the premises *(51)* and increased purchasing off the premises *(52)*. New products may be targeted at vulnerable segments of the population, for example, flavoured alcoholic beverages that have led to increased drinking among adolescents *(53)*.
8.1 Server training	Serving practices can be modified, such as refusing service to intoxicated customers and promoting food instead of drinks *(11)*.
8.2 Health warning labels	Health warning labels do not have an impact on drinking behaviour per se but may affect intervening variables such as the intention to change consumption and a willingness to intervene regarding drinking by others *(54)*.

Pricing policies and marketing of alcoholic beverages

The basic concept behind pricing policies is to constrain consumers' ability or willingness to purchase alcohol. It has been demonstrated consistently that drinkers reduce their consumption in response to price increases on alcoholic beverages *(50,55)*. This effect is observed for beer, wine and spirits, albeit to differing degrees depending on the characteristics of alcohol consumption in a country. The type of beverage with the dominant market share tends to be less affected by price fluctuations *(56)*. Overall, the results of two meta-analyses suggest that an average 5% reduction in per capita alcohol consumption is achieved for each 10% increase in price *(57,58)*. The converse is also shown to be true: in Finland, decreases in excise duties coupled with the removal of travellers' tax-free imports drove consumption up in 2004 by approximately 10% *(59)*. Importantly, the literature indicates that price changes have an impact on heavy drinkers and can lead to reductions in alcohol-related harm, including from liver cirrhosis and injuries *(50,60)*.

The EAPA composite indicators seek to capture differences in the affordability of alcohol and not alcohol prices per se. A new measure of affordability, the price index formula, was created to compare countries on the basis of alcohol prices in relation to income. This is an offshoot of the approach used by Brand et al. *(31)*, although it is unclear which GDP measure they used. Given that their project focused on a relatively homogenous group of high-income member countries of the OECD, it might be argued that different GDP estimates would have given similar results. In contrast, the present WHO project includes countries with divergent wealth and welfare conditions. Adjusting for disparities in the cost of living through the use of gross national income at PPP enabled fairer cross-country comparisons. The price index was calculated separately for beer, wine and spirits, and an overall score for the affordability indicator was determined using the banding approach described in the section on methods. However, the drawback of this approach is that it does not account for potential cross-beverage substitution. Substitution occurs when drinkers react to the increased price of beverages in one category by consuming more of different alcohol products. There is evidence of partial substitution between different types of alcohol, beverages of different quality and even between products sold in off-premise and on-premise settings *(61,62)*. Since the availability of low-cost alternatives encourages substitution *(61)*, a reasonable way forward might be to advocate that prices be high across the board. A modified scoring scheme based on this principle would have a final score that is wholly or mostly attributable to the beverage type with the cheapest price index rather than representing the average affordability of all beverage types. This methodological option may be explored in the future provided that there is stronger evidence behind cross-beverage substitution and improved capabilities among Member States for the accurate monitoring of alcohol prices.

There is a convincing body of evidence that connects alcohol marketing to undesirable drinking behaviour among young people. Systematic reviews of longitudinal studies have established that alcohol advertising induces earlier initiation of drinking and influences adolescents who already drink to increase the volume and intensity of their alcohol consumption *(53,63)*. Thus, restrictions on marketing activities are most likely to reduce alcohol-associated harm by modulating drinking patterns among children and teenagers. One study estimated that a complete alcohol advertising ban would bring about a 16.4% decrease in alcohol-attributable mortality in the United States through reductions in drinking prevalence among young people *(64)*. At the population level, aggregate and econometric analyses have found that alcohol advertising exerts only weak positive effects on total alcohol consumption in the short term *(58)*. Nevertheless, marketing plays a crucial role in shaping social attitudes towards drinking. For instance, a holiday was offered as a competition reward during a promotional campaign for beer in New Zealand, with the slogan: "the best weekend you'll never remember!" *(65)*. Such messages serve tacitly to normalize and even glamorize the practice of drinking to intoxication, thereby counteracting other health promotion efforts that discourage heavy drinking *(11)*. Even if an immediate reduction in consumption is not seen following the implementation of marketing restrictions, however, it is plausible that there are other long-term benefits such as a gradual weakening of the power of the alcohol industry to alter drinking norms *(66)*.

Marketing has emerged as one of the most challenging aspects of alcohol control because of the pervasiveness of alcohol advertising and promotion, which continue to evolve to include new media and technologies. The present WHO project includes marketing indicators in the four areas of advertising, product placement, event sponsorship and sales promotion so as to reflect the rapidly expanding repertoire of marketing-oriented activities. A total of 10 different platforms are considered under advertising restrictions (indicator 6.1). This is in line with current trends suggesting that television commercials are increasingly being replaced by novel forms of online advertising. Indeed, the major alcohol companies have been allocating more of their marketing budget to non-traditional projects such as social media campaigns *(53)*. Owing to the dearth of systematic research into the impact of various marketing strategies, it is unclear whether certain media outlets should be regulated more stringently. In the absence of any reason to believe that some platforms should be prioritized over others, the banding approach was adopted to reflect the general state of affairs in a country. The scoring system also assumes that binding restrictions are preferable to industry self-regulation. It has been shown time and again that voluntary codes are easily flouted and self-regulating bodies are ineffective in protecting young people from irresponsible marketing practices *(39,67,68)*.

RESULTS

Scoring scheme

The finalized scoring scheme comprises 34 SIs categorized in the 10 action areas of the EAPA (Table 9). Most of the SIs encompass multiple policy variables. Detailed scoring rubrics showing the composition of each SI are presented in Annex 2.

Table 9. Overview of scoring scheme for the EAPA composite indicators

Indicators		Maximum raw score	Multiplier level	Weighted score
1.	*Leadership, awareness and commitment*			
1.1	National policy on alcohol	4	3	12
1.2	Definition of alcoholic beverage	1	2	2
1.3	Definition of standard drink	1	1	1
1.4	Awareness activities	4	2	8
		Total possible points (after weighting)		*23*
2.	*Health services' response*			
2.1	Screening and brief interventions for harmful and hazardous alcohol use	10	3	30
2.2	Special treatment programmes	4	2	8
2.3	Pharmacological treatment	4	3	12
		Total possible points (after weighting)		*50*
3.	*Community and workplace action*			
3.1	School-based prevention and reduction of alcohol-related harm	4	2	8
3.2.	Workplace-based alcohol problem prevention and counselling	6	2	12
3.3	Community-based interventions to reduce alcohol-related harm	7	2	14
		Total possible points (after weighting)		*34*
4.	*Drink–driving policies and countermeasures*			
4.1	Maximum legal BAC limit when driving a vehicle	5	5	25
4.2	Enforcement using sobriety checkpoints	3	3	9
4.3	Enforcement using random breath-testing	4	4	16
4.4	Penalties	4	4	16
		Total possible points (after weighting)		*66*
5.	*Availability of alcohol*			
5.1	Lowest age limit for alcohol service on the premises and sale of alcohol for consumption off the premises	4	4	16
5.2	Control of retail sales	4	3	12
5.3	Restrictions on availability by time	4	3	12
5.4	Restrictions on availability by place	4	3	12
5.5	Restrictions on sales at specific events	3	3	9
5.6	Alcohol-free public environments	11	3	33
		Total possible points (after weighting)		*94*
6.	*Marketing of alcoholic beverages*			
6.1	Legally binding restrictions on alcohol advertising	4	3	12
6.2	Legally binding restrictions on product placement	4	3	12
6.3	Legally binding restrictions on industry sponsorship for sporting and youth events	4	3	12
6.4	Legally binding restrictions on sales promotions by producers, retailers and owners of pubs and bars	4	3	12
		Total possible points (after weighting)		*48*

Table 9 cont.

Indicators		Maximum raw score	Multiplier level	Weighted score
7.	*Pricing policies*			
7.1	Adjustment of taxation level for inflation	4	3	12
7.2	Affordability of alcoholic beverages	4	4	16
7.3	Other price measures	14	3	42
		Total possible points (after weighting)		*70*
8.	*Reducing the negative consequences of drinking and alcohol intoxication*			
8.1	Server training	3	2	6
8.2	Health warning labels	5	2	10
		Total possible points (after weighting)		*16*
9.	*Reducing the public health impact of illicit alcohol and informally produced alcohol*			
9.1	Use of duty paid or excise stamps on alcohol containers	3	3	9
9.2	Estimates of unrecorded alcohol consumption	3	3	9
9.3	Legislation to prevent illegal production and sale of alcoholic beverages	6	2	12
		Total possible points (after weighting)		*30*
10.	*Monitoring and surveillance*			
10.1	National system for monitoring	23	3	69
10.2	National surveys	7	3	21
		Total possible points (after weighting)		*90*

Regional scores

EAPA composite indicators were calculated for all Member States in the European Region for which sufficient data were available. Country scores for each action area were rescaled (0–100) for ease of comparison. The mean and median scores for the Region, as well as the minimum and maximum scores observed, are presented in Table 10. The lowest score obtained was zero for all but two action areas: health services' response and drink–driving policies and countermeasures. None of the countries obtained the maximum possible points for health services' response, availability of alcohol or pricing policies.

Table 10. Descriptive statistics of EAPA composite indicators (scaled)

Action area	Mean	Median	Minimum observed	Maximum observed
Leadership, awareness and commitment	65	74	0	100
Health services' response	51	51	12	94
Community and workplace action	47	47	0	100
Drink–driving policies and countermeasures	78	85	12	100
Availability of alcohol	60	64	0	94
Marketing of alcoholic beverages	52	50	0	100
Pricing policies	23	20	0	66
Reducing the negative consequences of drinking and alcohol intoxication	29	31	0	100
Reducing the public health impact of illicit alcohol and informally produced alcohol	60	70	0	100
Monitoring and surveillance	52	62	0	100

The distribution of country scores by action area is presented in Fig. 2–11, histograms of scores for the action areas of the European action plan. In general, Member States performed relatively well in the domain of drink–driving policies and countermeasures. Many countries fared poorly in the areas of pricing policies and reducing the negative consequences of drinking and alcohol intoxication.

Fig. 2. Leadership, awareness and commitment (n=47)

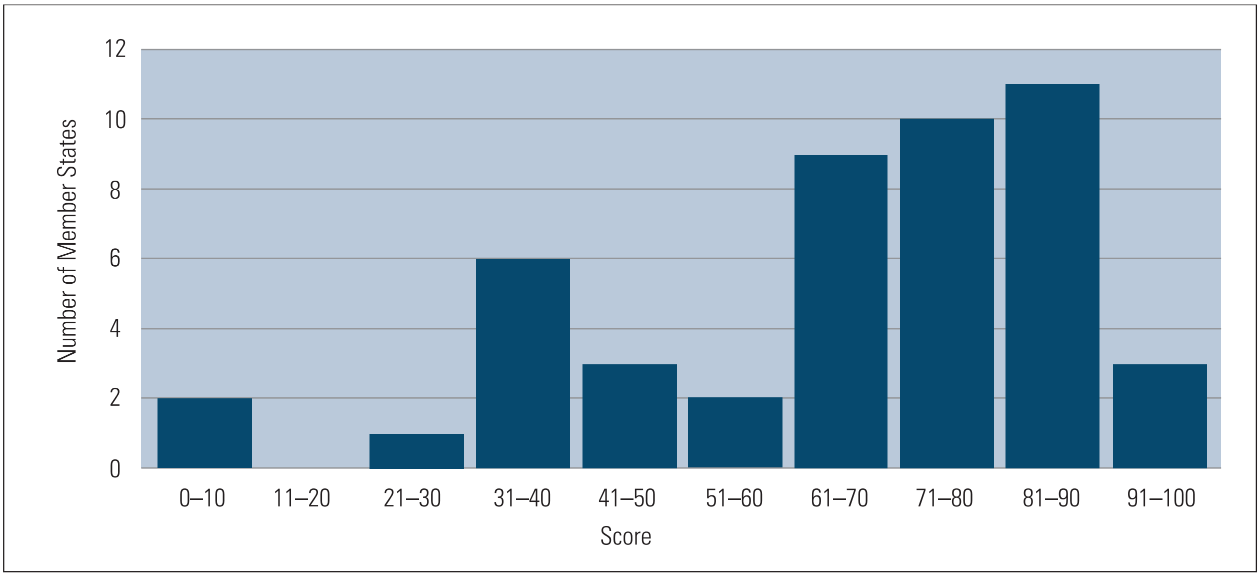

Fig. 3. Health services' response (n=34)

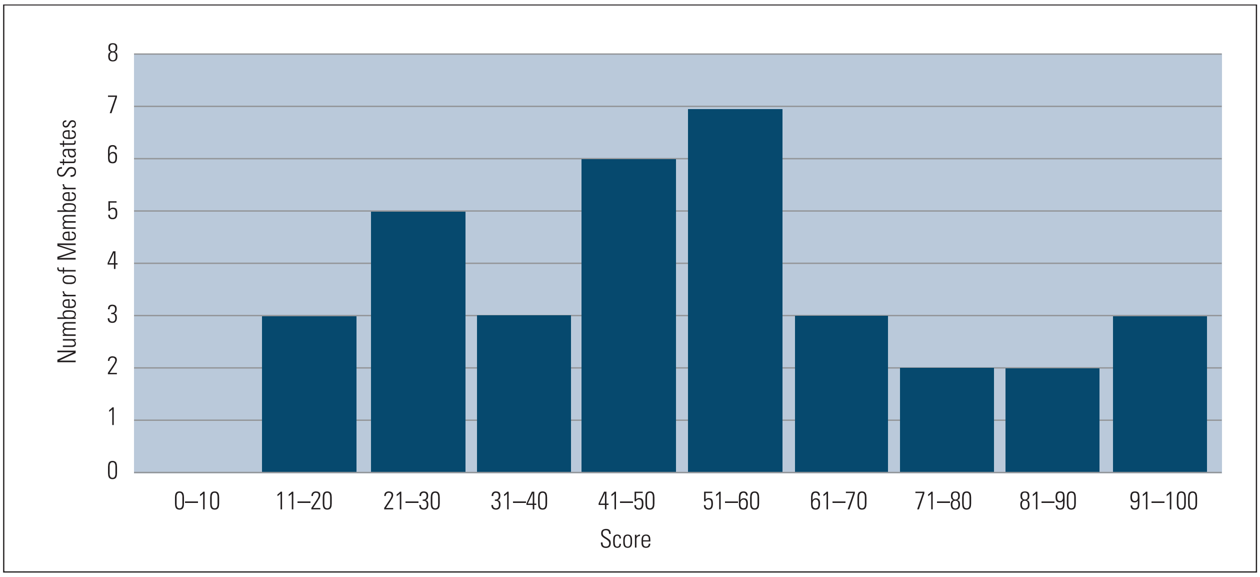

Fig. 4. Community and workplace action (n=47)

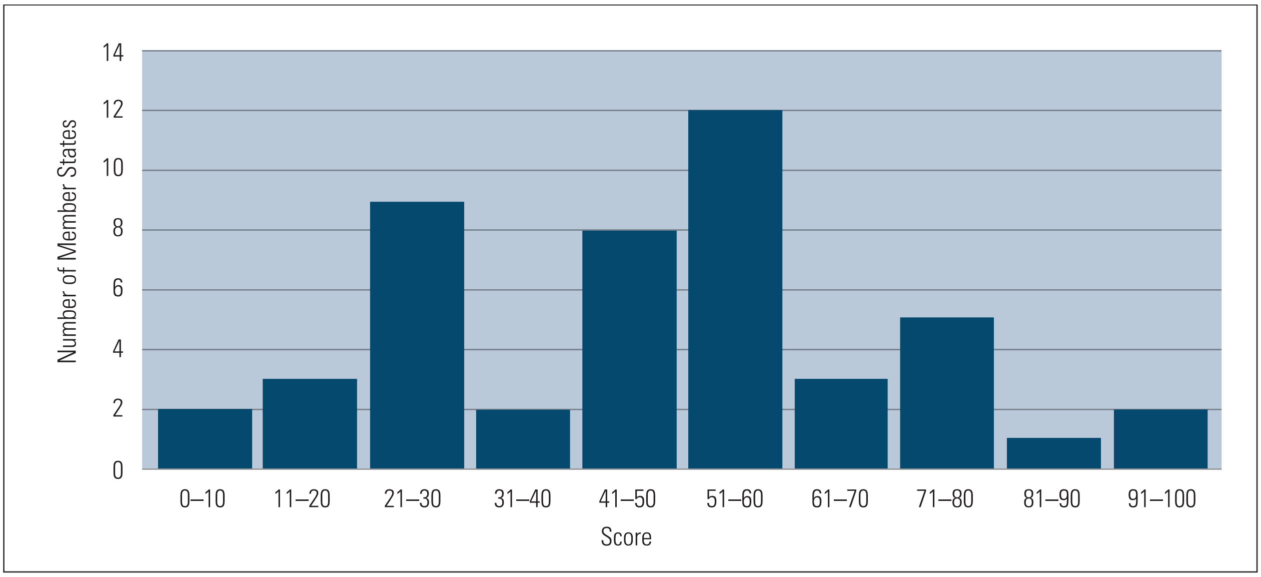

Fig. 5. Drink–driving policies and countermeasures (n=53)

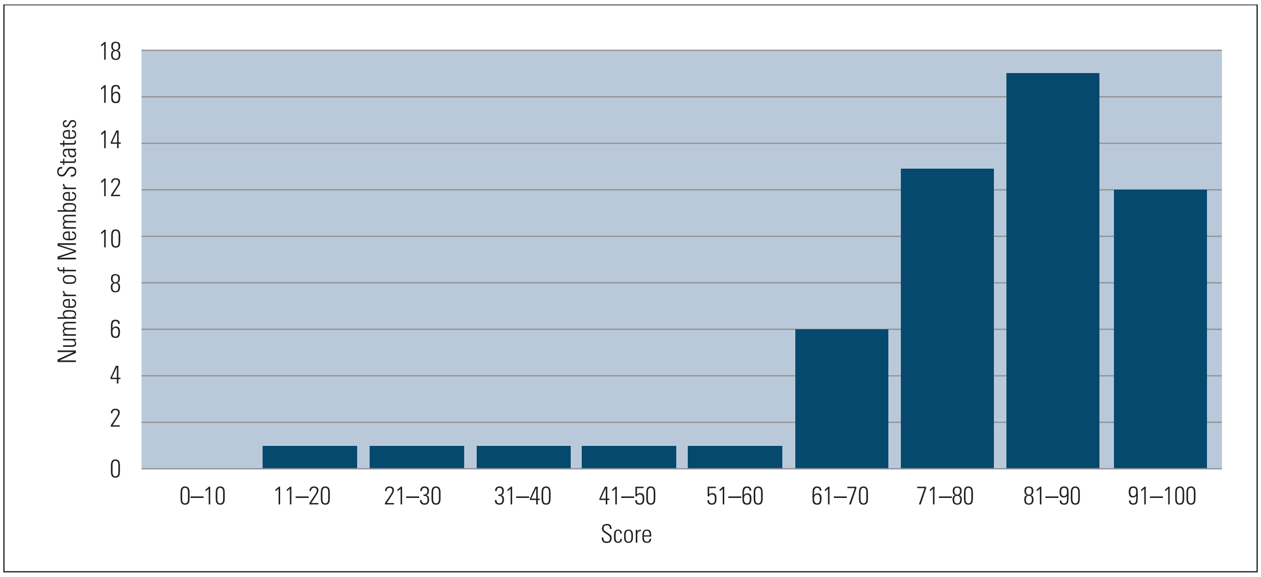

Fig. 6. Availability of alcohol (n=53)

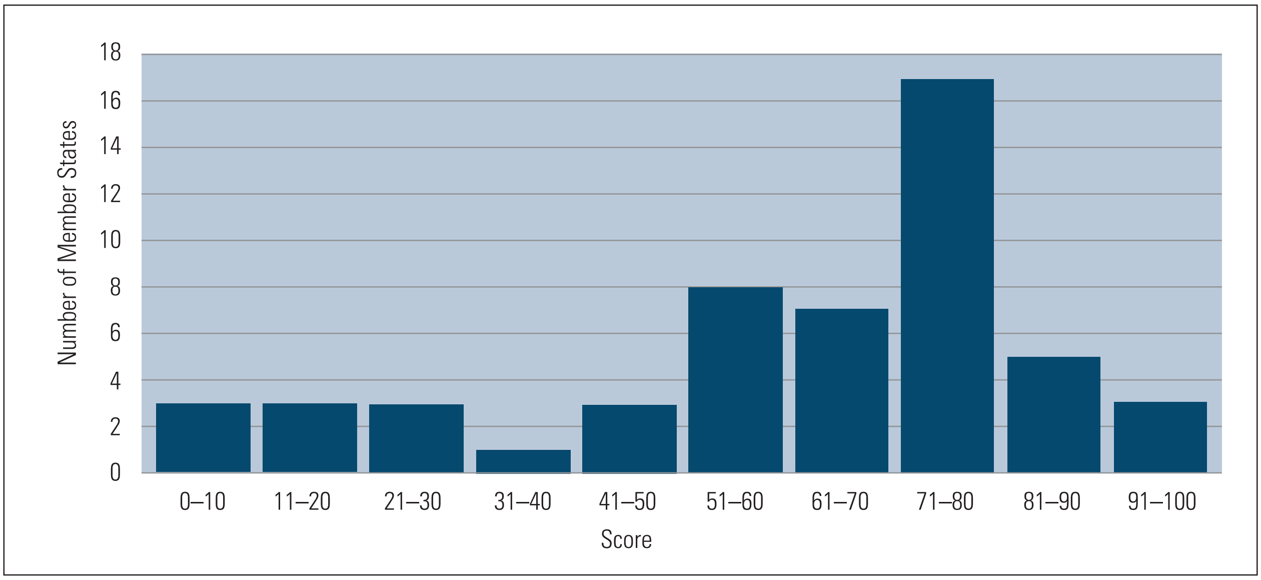

Fig. 7. Marketing of alcoholic beverages (n=53)

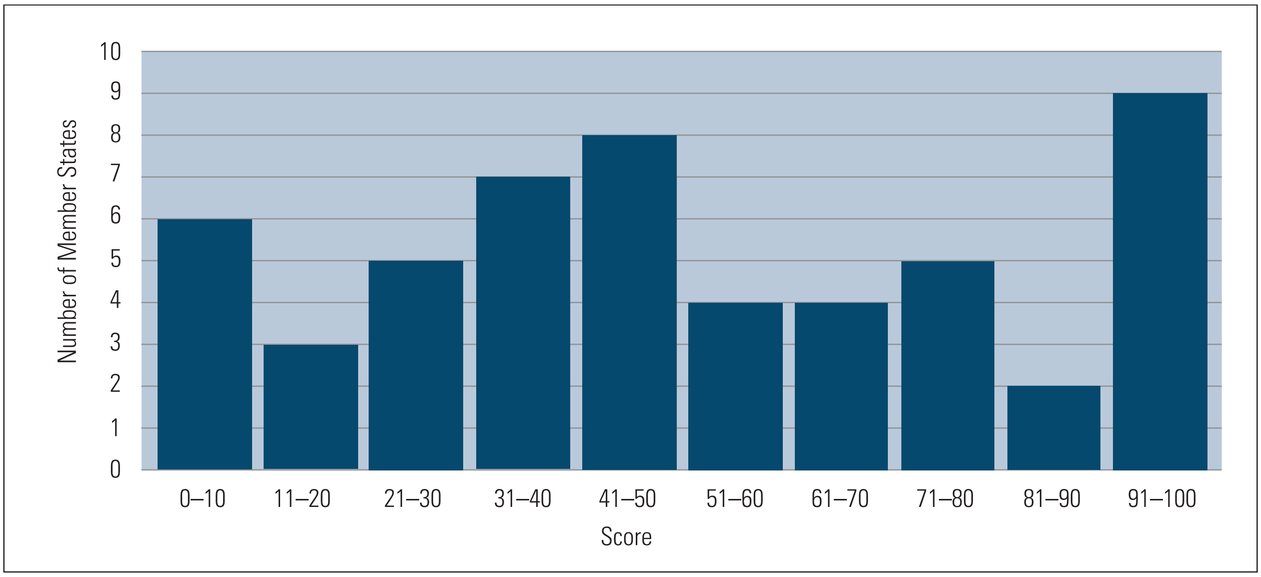

Fig. 8. Pricing policies (n=45)

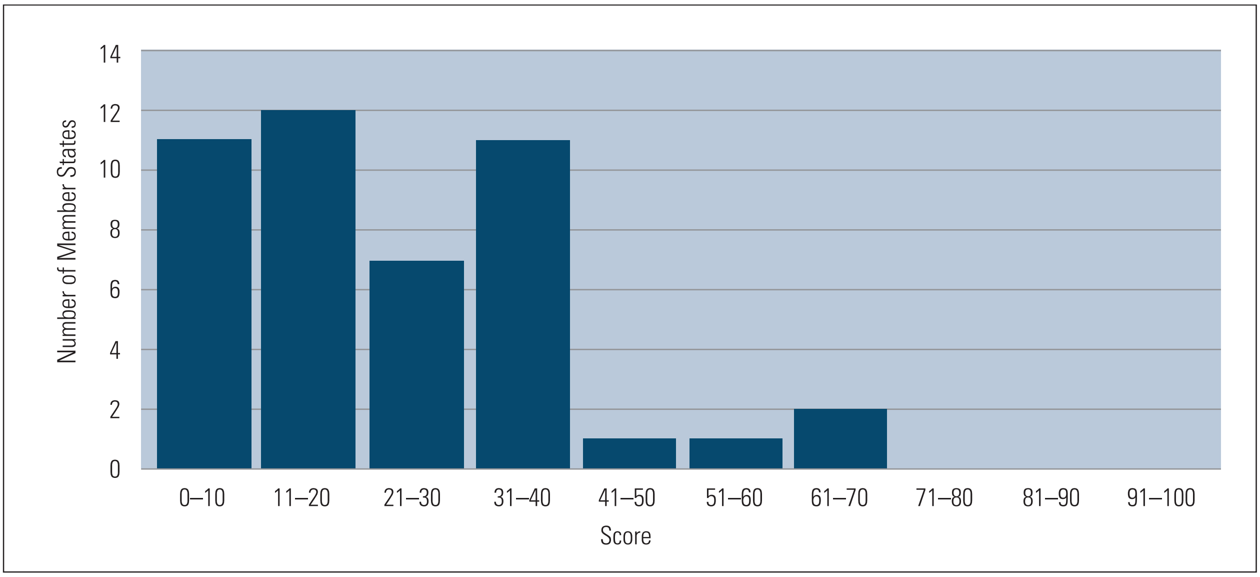

Fig. 9. Reducing the negative consequences of drinking and alcohol intoxication (n=52)

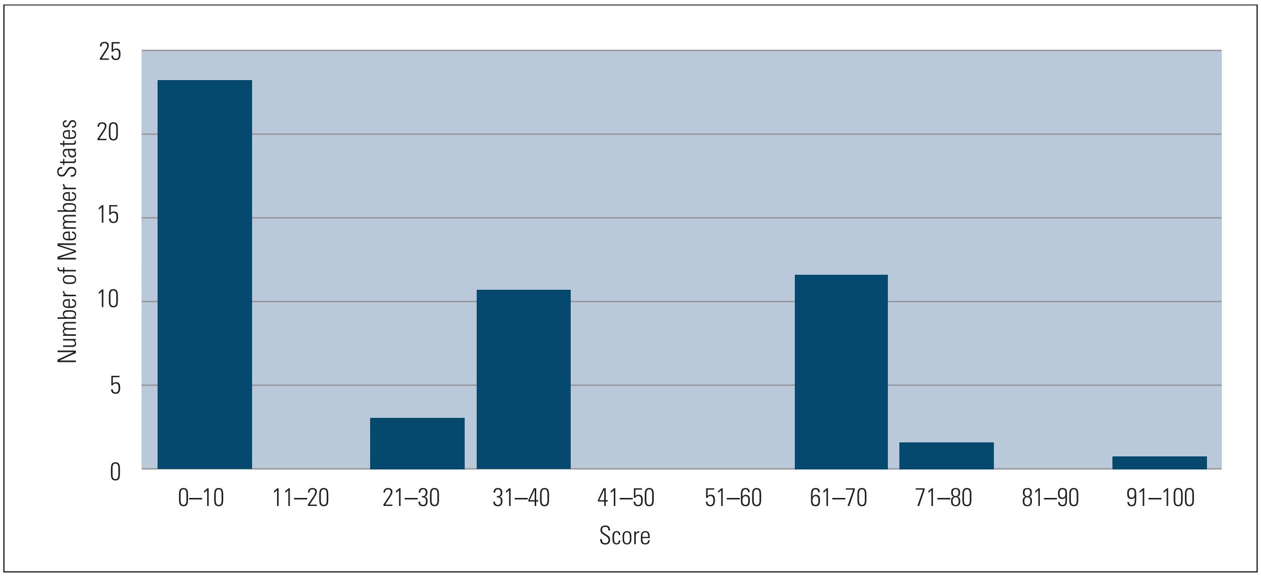

Fig. 10. Reducing the public health impact of illicit alcohol and informally produced alcohol (n=53)

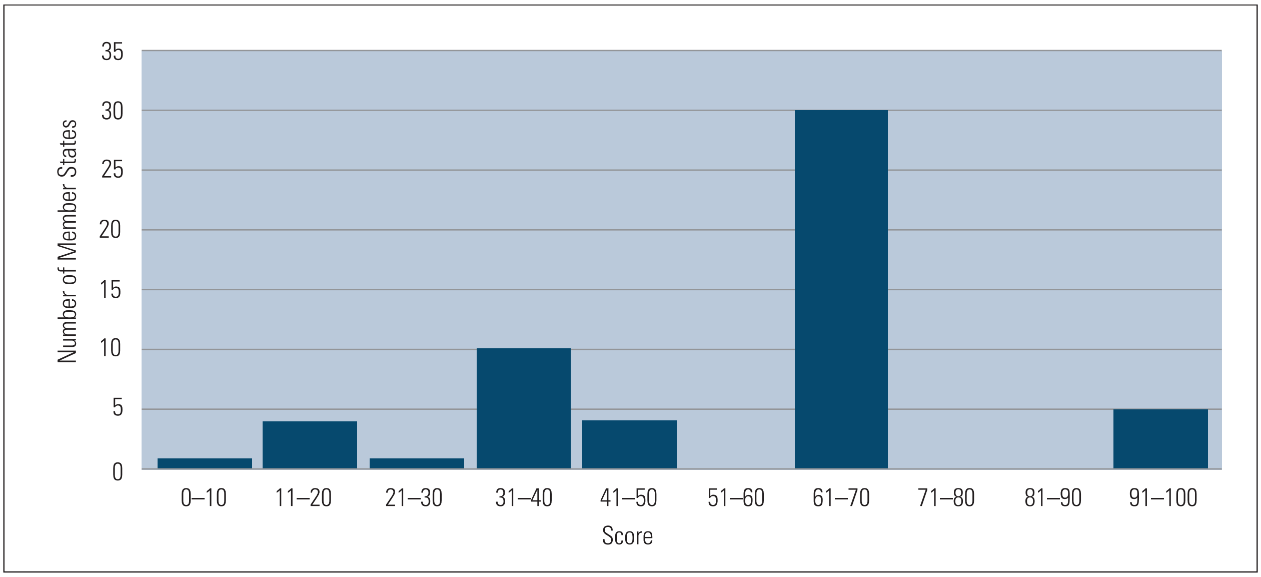

Fig. 11. Monitoring and surveillance (n=52)

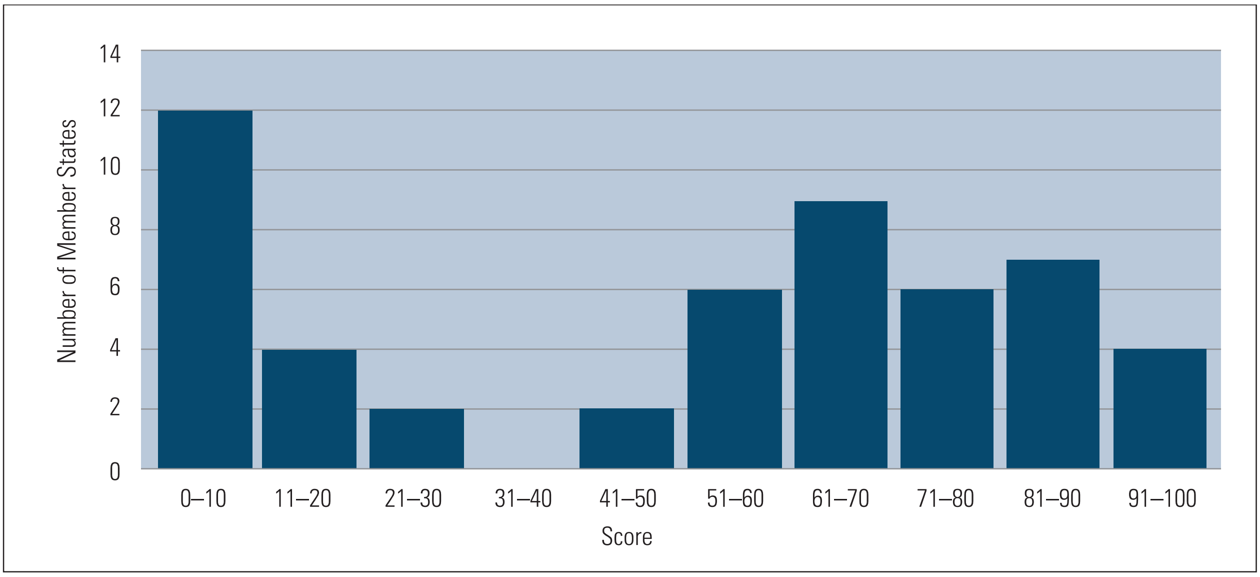

DISCUSSION

Summary of findings

The Regional Office developed these composite indicators with the aim of creating a tool to give guidance to Member States on the implementation of evidence-based alcohol policies, as described in the EAPA. The final scoring scheme is made up of 34 SIs spanning 10 action areas. Scores were computed and analysed for Member States in the European Region.

Improvements from previous composite indicators

This attempt to quantitatively compare the overall policy stance of national governments on alcohol, albeit not unprecedented, has been the most ambitious to date. Table 11 shows a comparison between the newly-developed EAPA composite indicators and several other assessments of alcohol policies. The EAPA composite indicators resemble most closely the scale that was developed as part of the AMPHORA project insofar as there is considerable overlap between the topic areas and the countries studied. However, several enhanced features of the EAPA composite indicators are worth noting. First, they measure a more diverse mix of policies compared to their counterparts. For example, evidence in favour of brief interventions has been accumulating over the years, but policies that target individual drinkers have hitherto been left out of alcohol policy metrics. Incorporating brief interventions into the EAPA composite indicators marks an important step forward in encouraging countries to leverage the untapped resource represented by health professionals and to use individual-level interventions to complement population-wide measures. Furthermore, even though certain policy domains were common to all three studies, differences become apparent when each domain is broken down to be examined. In the current WHO project, marketing restrictions comprise indicators on four fronts: advertising, sponsorship, product placement and sales promotion. In contrast, the AMPHORA scale evaluates only restrictions on advertising and sponsorship while the Alcohol Policy Index considers only restrictions on advertising. Second, the WHO project is the first to systematically analyse the alcohol policy situation in the countries of the former Union of Soviet Socialist Republics. These countries have the highest APC, proportion of heavy drinkers and alcohol-attributable deaths in the European Region *(4,6,16)*. In fact, the fifth PDS level was created especially to describe the characteristic drinking pattern seen in these countries, where there is a substantial number of deaths due to alcohol poisoning induced by binge drinking *(6)*. In short, the EAPA composite indicators are able to offer a more complete picture of the European alcohol policy landscape by virtue of their increased breadth (more indicators) and depth (more details for each indicator), as well as novel sample characteristics (a larger sample with more diverse countries).

Table 11. Comparison of EAPA composite indicators with three policy scoring projects

Study	EAPA composite indicators	AMPHORA scale[a]	Alcohol Policy Index[b]	Underage alcohol policies[c,d]
Year	2016	2012	2007	2012
Sample	53 Member States in the European Region	33 European countries	30 member countries of the OECD	50 cities in California, the United States
Policy domains	10 action areas:<br>• availability of alcohol<br>• monitoring and surveillance<br>• pricing policies<br>• leadership, awareness and commitment<br>• marketing of alcoholic beverages<br>• drink–driving policies and countermeasures	7 domains:<br>• control of production, retail sale and distribution of alcoholic beverages<br>• alcohol taxation and price<br>• age limits and personal control<br>• control of drink–driving	5 domains:<br>• motor vehicles<br>• physical availability<br>• alcohol prices<br>• drinking context<br>• alcohol advertising	8 ordinances:<br>• conditional use permits<br>• deemed approved ordinances<br>• regulations on outdoor advertising<br>• regulations on public drinking<br>• responsible beverage service<br>• social host policies

Table 11 cont.

Study	EAPA composite indicators	AMPHORA scale[a]	Alcohol Policy Index[b]	Underage alcohol policies[c,d]
	• health services' response<br>• community and workplace action<br>• reducing the public health impact of illicit alcohol and informally produced alcohol<br>• reducing the negative consequences of drinking and alcohol intoxication	• control of advertising, marketing and sponsorship of alcoholic beverages<br>• public policy<br>• starting points[e]		• special outdoor events policies<br>• regulations on window advertising
Data sources	EISAH 2012; RSUD 2014; with additional input from country experts and data from 2015 WHO global questionnaire on progress in alcohol policy	Questionnaire completed using data from EISAH 2011, with additional input from country experts	Published reports, databases maintained by WHO and individual countries (data for 2000–2005)	Legal data obtained from the website and city clerk of each city

[a] Karlsson et al. *(32)*.
[b] Brand et al. *(31)*.
[c] Thomas et al. *(69)*.
[d] Ordinances were analysed separately and not merged into a single score.
[e] Qualitative section without any scores.

Policy interactions

Many of the policies in the EAPA composite indicators are in fact mutually reinforcing and can lead to synergistic benefits. A positive association has been detected between the geographical density of establishments licensed to sell alcohol and the rate of drink–driving among young people, suggesting that policies aimed at limiting the density of outlets may bring about additional advantages such as a reduction in drink–driving incidents *(70)*. Conversely, the absence of some policies can undermine the effectiveness of others. For example, raising the price of vodka in the Russian Federation did not produce the expected reduction in total alcohol consumption as consumers were simply driven to purchase cheap illegal moonshine *(71)*. This underscores the importance of keeping a check on illicit alcohol so that taxation of licit alcohol can be effective. Ultimately, the success of a national alcohol strategy is determined by the net output from this dynamic interplay of policy factors (as well as other contextual factors), so different combinations of policies can be expected to produce different results. It was not, however, feasible for policy interactions to be built into the scoring system owing to a lack of empirical data in this area.

Robustness of the EAPA composite indicators

A thorough sensitivity analysis should be carried out in the future and several aspects of the composite indicators investigated. First, any questionable rules underlying the SIs should be varied and tested. Using the affordability of alcoholic beverages (indicator 7.2) as an example, and keeping in mind potential cross-beverage substitution, the lowest price level instead of the average price level could be used to determine the final score. Moreover, the price levels are demarcated using arbitrary cut-off points, and adjustment of these thresholds may lead to considerable changes in the scoring outcome. Second, a different set of policy weights could be used. Alternatively, country-specific weights may be derived using data envelopment analysis, a technique which seeks to maximize the score of each country vis-à-vis all other countries. This approach was used by Brand et al. *(31)* to counter possible criticisms that countries may have regarding biases in the policy weights. Third, missing data could be dealt with using more sophisticated methods such as regression imputation or nearest-neighbour imputation.

Strengths and limitations of the EAPA composite indicators

The EAPA composite indicators can be generated at regular intervals throughout the lifespan of the European action plan (2012–2020), probably in synchrony with WHO surveys, such that it is possible to quantitatively monitor the progress of individual countries. These periodic "report cards" accord recognition to role models while motivating countries that are lagging behind to make good on their commitment. The EAPA composite indicators give guidance for politicians to identify areas of alcohol policy where a Member State has low scores. Furthermore, they offer an important sense of regional solidarity – "countries across the world are seen to move in step. That is perhaps the greatest reassurance which politicians can have when adopting potentially unpopular policies" *(72)*. The EAPA composite indicators are more suited to advocacy than previous attempts because the project is tied explicitly to a framework that has been endorsed by all 53 Member States in the European Region. Nevertheless, it would be useful in the future to consider feedback from representatives of Member States as this would help to establish the face validity of the composite indicators, that is, the "acceptance by stakeholders that the measure is useful and valid" *(34)*.

A critical component of advocacy is the process of communication with stakeholders. Currently, regular status reports are produced by WHO both regionally *(73,74)* and globally *(4,75,76)* to describe trends in alcohol consumption, harm and policy responses. These reports are a valuable trove of information and allow the whole range of EISAH indicators to be scrutinized. The EAPA composite indicators complement this by presenting the same information in a more compact and digestible form and would be particularly relevant for communicating with laypersons, including in the media and the general public.

A weakness of the EAPA composite indicators is, however, that they take reported legislation and policies at face value although these may not actually be translated into action. Enforcement can make or break a potentially successful policy. In the United States, for example, despite the minimum legal drinking age of 21 years, a national survey revealed that more than 90% of underage students were able to break their college alcohol rules without being subjected to sanctions *(77)*. In Brazil, on the other hand, the positive impact of a lower BAC limit on reducing traffic fatalities has been attributed in part to an intensification of police enforcement *(78)*. In the EISAH surveys, national experts were requested to provide policy enforcement ratings. Marked changes in ratings over time may be indicative of genuine changes in enforcement activity. However, the subjectivity of data obtained in this manner might introduce bias and complicate the interpretation of cross-country comparisons. These enforcement ratings were, therefore, deemed too unreliable to be directly integrated into the EAPA composite indicators. A proxy enforcement measure used by Thomas et al. *(69)* was the amount of competitive state funding secured by each city in California (United States) for the enforcement of alcohol policies. Although this is an innovative approach, it is not easily transferable to studies involving international comparisons. Thus, the problem of incorporating objective enforcement measures into the EAPA composite indicators remains intractable at present and highlights a research gap in the alcohol policy field.

The EAPA composite indicators only register planned interventions delivered through official channels. Yet the "powerful informal rules and controls of civil society governing drinking and intoxication behaviour" are just as important in preventing alcohol-related problems *(79)*. For instance, the increased underage drinking observed with a higher density of alcohol outlets has been shown to be attenuated in environments with higher collective efficacy and informal control *(80)*. Paradoxically, normative attitudes towards alcohol appear to be inversely correlated with how strictly alcohol is governed. Countries with a more relaxed regulatory climate for alcohol exhibit a lower tolerance of drunkenness *(81)*. Besides informal controls, alcohol consumption at the population level is also affected by structural changes in society. For example, alcohol consumption in Italy started to fall even before any official alcohol policies were put in place. This was later attributed to urbanization and changes in the organization of work *(82)*. Other structural pressures which have a potential impact on alcohol consumption include changes in family structure and gender roles, a proliferation of motorized vehicles, an influx of immigrants and economic booms and recessions. Interestingly, the same structural changes have resulted in different trends in consumption in different societies *(66,83)*. It is, therefore, important to recognize that alcohol consumption is embedded in a complex web of personal, cultural and structural factors, not all of which are directly amenable to modification through formal policies. Nevertheless, this does not detract from the need for countries to implement evidence-based prevention policies within these contextual constraints.

An additional limitation of this project is the large amount of missing data for some indicators, particularly in the action area of health services' response where scores for 18 countries could not be calculated due to insufficient data. This was largely the result of missing data for SI 2.1: screening and brief interventions for harmful and hazardous alcohol

use. Specifically, many respondents were unable to provide an estimate of the proportion of primary health care services and the proportion of antenatal services that have implemented screening and brief interventions for harmful and hazardous substance use at the national level. The lack of available data point to the need for improved monitoring of these programmes at the national level.

A summary of the strengths and limitations of the EAPA composite indicators is given in Table 12.

Table 12. Strengths and limitations of the EAPA composite indicators

Strengths	Limitations
• The role of governments in reducing population exposure to modifiable risk factors is emphasized.<br>• Political accountability is promoted.<br>• Regional/global solidarity is fostered.<br>• A rounded evaluation of national alcohol strategies is provided.<br>• A big picture for each overarching policy area is presented which is easier to grasp than separate trends across many different indicators.<br>• Comparisons between countries are facilitated.<br>• Monitoring of a country's progress over time is facilitated.<br>• Communication with stakeholders is simplified.	• Enforcement of policies is not measured.<br>• Informal controls and contextual determinants of alcohol consumption are not accounted for.<br>• Some aspects of the methodology (such as policy weights) are potentially contentious.<br>• Data for some indicators (such as pricing estimates) are less reliable.<br>• There are large amounts of missing data in some policy areas (such as screening and brief interventions).<br>• The details of a composite indicator may need to be adjusted as newer research evidence becomes available.<br>• Aggregated information does not reflect subnational variations in alcohol policies.[a]<br>• Summary measures are prone to being misinterpreted.

[a] For the United Kingdom, points were awarded if the policy applied to two or more of the nations.

Future work

Certain countries may feel that they are unfairly penalized by the EAPA composite indicators, which prescribe a set of universal policy standards. If existing policy provisions and sociocultural pressures have been enough to suppress consumption in a country, the government might find it overly heavyhanded to implement the additional controls set out in the EAPA composite indicators. It is indeed a valid concern that interventions ought to be proportionate to the magnitude of the alcohol problem *(72)*. This could, perhaps, be circumvented by incorporating outcome measures. Countries could be given points based on the absolute APC level, the PDS and the alcohol-attributable mortality rate and whether these figures have improved, worsened or stabilized. A country with falling alcohol consumption, a lower PDS and fewer alcohol-attributable deaths would start off with higher scores. It follows that countries performing poorly on these outcome indicators should chalk up more policy points in order to be on a par with countries that have a more favourable baseline situation (Fig. 12). By including outcome measures, some of the limitations discussed above are addressed indirectly. This is because the consumption level reflects to some extent the strength of informal controls and how rigorously existing policies are enforced. Composite indicators encapsulating both policy input and health outcomes are, however, harder to interpret, and greater care must be taken when explaining the results of such an analysis.

Another potential development of the EAPA composite indicators is to marry them with the tobacco control scale *(84)* so as to stimulate concerted efforts to prevent noncommunicable diseases. The tobacco control scale is a composite indicator that quantifies country-level tobacco control activity in Europe. Alcohol and tobacco are interlinked on many levels and go hand-in-hand as key drivers of the global epidemic of noncommunicable diseases *(85)*. Tobacco use is associated with more frequent and longer drinking episodes and smokers are more likely to meet the criteria for binge drinking, hazardous drinking and alcohol use disorder diagnoses *(86)*. In the same vein, policies aimed primarily at reducing smoking may actually help to bring down alcohol consumption. For instance, in Ireland alcohol sales in bars decreased by around 4.6% after a comprehensive smoking ban came into force *(87)*. Thus, tobacco control and alcohol control are mutually reinforcing activities. A WHO global monitoring framework has been set up recently with voluntary targets for reductions in both alcohol and tobacco consumption *(85)*. Concurrent use of the EAPA composite indicators and the tobacco control scale in policy and research could be one way of encouraging collaboration between public health officials in both fields. While beyond the scope of this project at the moment, other relevant composite indicators

Fig. 12. Hypothetical example of two countries with identical scores if an EAPA composite indicator is modified to include outcome measures[a]

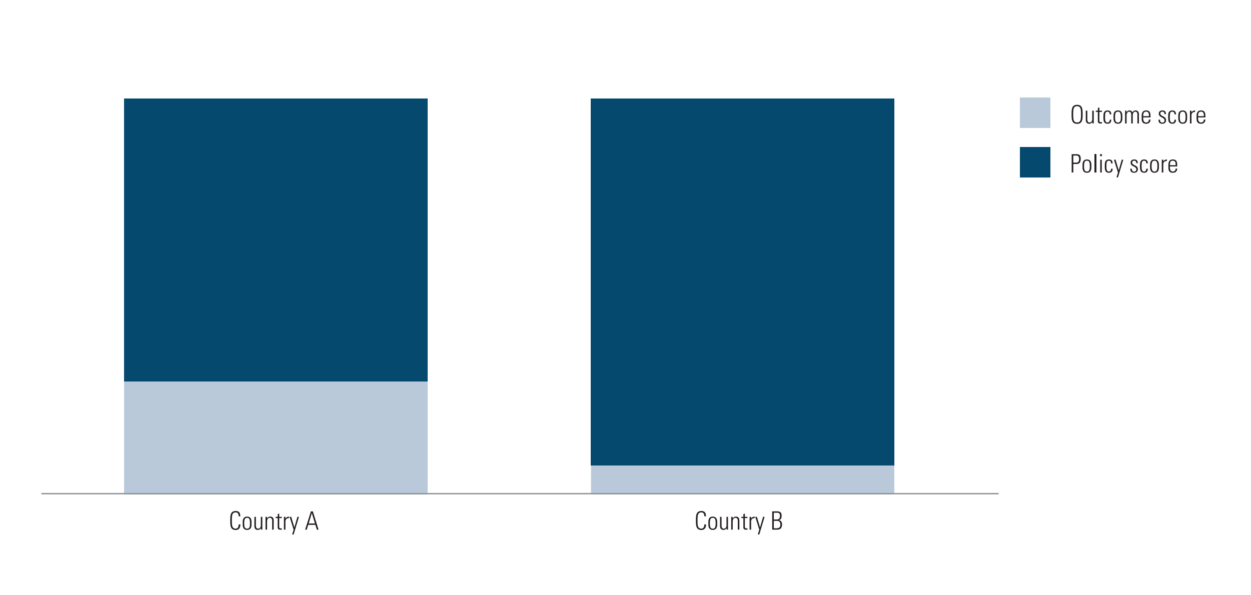

[a] The assumption is that it is acceptable for countries reporting lower alcohol consumption and harm (country A) to have a smaller or less stringent set of policies.

(such as obesity prevention) could be developed and added to the list in order to obtain a holistic assessment of each country's progress in tackling the major risk factors for noncommunicable diseases.

Although this project focuses on countries in the European Region, the EAPA composite indicators could potentially be adapted to other regions. There are, however, two important caveats. Most of the evidence underpinning the EAPA composite indicators was derived from research conducted in high-income countries *(68)*. The cost–effectiveness of the recommended strategies is also expected to vary considerably between different geographical subregions *(88)*. While it would be beneficial to accrue more empirical information on the feasibility, effectiveness and acceptability of these interventions in low-resource settings, as other authors have pointed out *(68,89)*, the overwhelming detriments of alcohol justify immediate precautionary action. The EAPA composite indicators provide a valuable framework for developing countries to review their policy response and take steps toward curtailing the proliferation of alcohol.

CONCLUSION

Composite indicators tied to the European action plan were developed to measure not only the presence of a range of alcohol policies, but also the extent to which they meet recommended standards of strictness and comprehensiveness. This was done via a stepwise approach to selecting, scaling, weighting and recoding relevant policy variables. The EAPA composite indicators can be used for performance benchmarking, monitoring trends over time, comparing policy options and communicating with stakeholders and the public. Further work can be done to ascertain the robustness of the composite indicators and their political acceptability. The European Region has the highest alcohol consumption and alcohol-attributable mortality in the world, and it is envisaged that the EAPA composite indicators will spur governments on to remedy this situation.

REFERENCES

1. Schmidt LA, Room R. Alcohol and the process of economic development: contributions from ethnographic research. Int J Alcohol Drug Res. 2012;1:41–55.
2. European action plan to reduce the harmful use of alcohol 2012–2020. Copenhagen: WHO Regional Office for Europe; 2012 (http://www.euro.who.int/__data/assets/pdf_file/0008/178163/E96726.pdf?ua=1, accessed 8 February 2017).
3. Lim SS, Vos T, Flaxman AD, Danaei G, Shibuya K, Adair-Rohani H et al. A comparative risk assessment of burden of disease and injury attributable to 67 risk factors and risk factor clusters in 21 regions, 1990-2010: a systematic analysis for the Global Burden of Disease Study 2010. Lancet. 2012;380(9859):2224–60 (http://www.ncbi.nlm.nih.gov/pubmed/23245609, accessed 8 February 2017).
4. Global status report on alcohol and health 2014. Geneva: World Health Organization; 2014 (http://www.who.int/substance_abuse/publications/global_alcohol_report/msb_gsr_2014_1.pdf?ua=1, accessed 8 February 2017).
5. International classification of diseases and related health problems, 10th revision [website]. Geneva: World Health Organization; 2010 (http://apps.who.int/classifications/icd10/browse/2010/en, accessed 8 February 2017).
6. Rehm J, Mathers C, Popova S, Thavorncharoensap M, Teerawattananon Y, Patra J. Global burden of disease and injury and economic cost attributable to alcohol use and alcohol-use disorders. Lancet. 2009;373(9682):2223–33 (http://linkinghub.elsevier.com/retrieve/pii/S0140673609607467, accessed 8 February 2017).
7. Rehm J, Roerecke M. Reduction of drinking in problem drinkers and all-cause mortality. Alcohol Alcohol. 2013;48:509–13 (http://www.ncbi.nlm.nih.gov/pubmed/23531718, accessed 8 February 2017).
8. Rehm J, Room R, Graham K, Monteiro M, Gmel G, Sempos CT. The relationship of average volume of alcohol consumption and patterns of drinking to burden of disease: an overview. Addiction. 2003;98:1209–28 (http://www.ncbi.nlm.nih.gov/pubmed/12930209, accessed 8 February 2017).
9. Corrao G, Rubbiati L, Bagnardi V, Zambon A, Poikolainen K. Alcohol and coronary heart disease: a meta-analysis. Addiction. 2000;95:1505–23 (http://www.ncbi.nlm.nih.gov/pubmed/11070527, accessed 8 February 2017).
10. Alcohol in the European Union: Consumption, harm and policy approaches, Copenhagen: WHO Regional Office for Europe; 2012 (http://www.euro.who.int/__data/assets/pdf_file/0003/160680/e96457.pdf?ua=1, accessed 8 February 2017).
11. Babor TF, Caetano R, Casswell S, Edwards G, Giesbrecht N, Graham K. Alcohol: no ordinary commodity. Research and public policy. 2nd ed. Oxford: Oxford University Press; 2010.
12. Anderson P, Baumberg B. Alcohol in Europe. A public health perspective. London: Institute of Alcohol Studies; 2006 (http://ec.europa.eu/health/ph_determinants/life_style/alcohol/documents/alcohol_europe.pdf, accessed 8 February 2017).
13. Loring B. Alcohol and inequities. Guidance for addressing inequities in alcohol-related harm. Copenhagen: WHO Regional Office for Europe; 2014 (http://ec.europa.eu/health/social_determinants/docs/policybrief_alcohol_en.pdf, accessed 8 February 2017).
14. Blas E, Kurup AS, editors. Equity, social determinants, and public health programmes. Geneva: World Health Organization; 2010 (http://whqlibdoc.who.int/publications/2010/9789241563970_eng.pdf, accessed 8 February 2017).
15. Beard E, Brown J, West R, Angus C, Brennan A, Holmes J et al. Deconstructing the Alcohol Harm Paradox: A Population Based Survey of Adults in England. PLoS ONE. 2016;11(9):e0160666.
16. Shield KD, Rylett M, Gmel G, Gmel G, Kehoe-Chan TA, Rehm J. Global alcohol exposure estimates by country, territory and region for 2005 – a contribution to the comparative risk assessment for the 2010 Global Burden of Disease Study. Addiction. 2013;108(5):912–22 (http://onlinelibrary.wiley.com/doi/10.1111/add.12112/abstract, accessed 8 February 2017).
17. Shield KD, Rylett M, Rehm J. Public health successes and missed opportunities. Trends in alcohol consumption and attributable mortality in the WHO European Region, 1990–2014. Copenhagen: WHO Regional Office for Europe; 2016 (http://www.drugsandalcohol.ie/26179/1/Public-health-successes-and-missed-opportunities-alcohol-mortality-19902014.pdf, accessed 8 February 2017).
18. Global strategy to reduce the harmful use of alcohol. Geneva: World Health Organization; 2010 (http://www.who.int/substance_abuse/msbalcstragegy.pdf, accessed 8 February 2017).

19. Outline of the process that led to the WHO Global strategy to reduce harmful use of alcohol [website]. Geneva: World Health Organization; 2016 (http://www.who.int/substance_abuse/activities/globalstrategy/en/, accessed 8 February 2017).
20. Rekve D. A global frame is already in place. Addiction. 2013;108(3):460–1 (http://onlinelibrary.wiley.com/doi/10.1111/j.1360-0443.2012.04070.x/full, accessed 8 February 2017).
21. Handbook on constructing composite indicators: methodology and user guide. Paris: Organisation for Economic Co-operation and Development; 2008 (http://www.oecd.org/std/leading-indicators/42495745.pdf, accessed 8 February 2017).
22. Bandura R. A survey of composite indices measuring country performance: 2008 update. New York (NY): United Nations Development Programme, Office of Development Studies; 2008 (http://web.undp.org/developmentstudies/docs/indices_2008_bandura.pdf, 8 February 2017).
23. Human Development Index (HDI) [website]. New York (NY): United Nations Development Programme; 2013 (http://hdr.undp.org/en/statistics/hdi/, accessed 8 February 2017).
24. Schwab K, editor. The global competitiveness report: 2012–2013. Geneva: World Economic Forum; 2012 (http://www3.weforum.org/docs/WEF_GlobalCompetitivenessReport_2012-13.pdf, accessed 8 February 2017).
25. Corruption Perceptions Index [website]. Berlin: Transparency International; 2014 (http://www.transparency.org/research/cpi/overview, accessed 8 February 2017].
26. Murray CJL, Lauer J, Tandon A, Frenk J. Overall health system achievement for 191 countries. Global Programme on Evidence for Health Policy discussion paper No. 28. Geneva: World Health Organization; 2000 (http://www.who.int/healthinfo/paper28.pdf, accessed 8 February 2017).
27. Gakidou E, Murray CJL, Frenk J. Measuring preferences on health system performance assessment. Global Programme on Evidence for Health Policy discussion paper No. 20. Geneva: World Health Organization; 2000 (http://www.who.int/healthinfo/paper20.pdf, accessed 8 February 2017).
28. Better Life Index [website]. Paris: Organisation for Economic Co-operation and Development; 2014 (http://www.oecdbetterlifeindex.org/, accessed 8 February 2017].
29. Naimi TS, Blanchette J, Nelson TF, Nguyen T, Oussayef N, Heeren TC et al. A new scale of the US alcohol policy environment and its relationship to binge drinking. Am J Prev Med. 2014;46(1):10–6.
30. Nelson TF, Xuan Z, Babor TF, Brewer RD, Chaloupka FJ, Gruenewald PJ. Efficacy and the strength of evidence of US alcohol control policies. Am J Prev Med. 2013;45(1):19–28.
31. Brand DA, Saisana M, Rynn LA, Pennoni F, Lowenfels AB. Comparative analysis of alcohol control policies in 30 countries. PLOS Medicine. 2007;4:e151 (http://journals.plos.org/plosmedicine/article?id=10.1371/journal.pmed.0040151, accessed 8 February 2017).
32. Karlsson T, Lindeman M, Osterberg E. Does alcohol policy-make any difference? Scales and consumption. In: Anderson P, Braddick F, Reynolds J, Gual A, editors. Alcohol policy in Europe: evidence from AMPHORA [e-book]. The AMPHORA project; 2012 (http://amphoraproject.net/w2box/data/e-book/Chapter%203%20-%20AM_E-BOOK_2nd%20edition%20-%20June%202013.pdf, accessed 8 February 2017).
33. Carragher N, Byrnes J, Doran CM, Shakeshaft A. Developing an alcohol policy assessment toolkit: application in the western Pacific. Bull World Health Organ. 2014;92:726–33 (http://www.who.int/bulletin/volumes/92/10/13-130708.pdf, accessed 8 February 2017).
34. Peterson ED, DeLong ER, Masoudi FA, O'Brien SM, Peterson PN, Rumsfeld JS et al. ACCF/AHA 2010 Position Statement on Composite Measures for Health Care Performance Assessment. A report of the American College of Cardiology Foundation/American Heart Association Task Force on Performance Measures (Writing Committee to Develop a Position on Composite Measures). Circulation. 2010;121:1780–91 (http://circ.ahajournals.org/content/121/15/1780, accessed 8 February 2017).
35. Jacobs R, Smith P, Goddard M. Measuring performance: an examination of composite performance indicators. York: Centre for Health Economics, University of York; 2004 (CHE Technical Paper Series 29; http://www.york.ac.uk/che/pdf/tp29.pdf, accessed 8 February 2017).
36. GNI per capita, PPP (current international $) [online database]. Washington (DC): World Bank; 2016 (http://data.worldbank.org/indicator/NY.GNP.PCAP.PP.CD, accessed 8 February 2017).
37. Human Development Reports. Andorra [web site]. New York (NY): United Nations Development Programme; 2015 (http://hdr.undp.org/en/countries/profiles/AND, accessed 8 February 2017).
38. Esser MB, Jernigan DH. Assessing restrictiveness of national alcohol marketing policies. Alcohol and Alcohol. 2014;49(5):557–62. https://www.ncbi.nlm.nih.gov/pubmed/25113175, accessed 6 April 2017).

39. Anderson P, Chisholm D, Fuhr DC. Effectiveness and cost-effectiveness of policies and programmes to reduce the harm caused by alcohol. Lancet. 2009;373(9682):2234–46 (http://www.ncbi.nlm.nih.gov/pubmed/19560605, accessed 8 February 2017).
40. Elder RW, Shults RA, Sleet DA, Nichols JL, Thompson RS, Rajab W. Effectiveness of mass media campaigns for reducing drinking and driving and alcohol-involved crashes: a systematic review. Am J Prev Med. 2004;27:57–65 (https://www.ncbi.nlm.nih.gov/pubmed/15212776, accessed 8 February 2017).
41. Evidence for the effectiveness and cost–effectiveness of interventions to reduce alcohol-related harm. Copenhagen: WHO Regional Office for Europe; 2009 (http://www.euro.who.int/__data/assets/pdf_file/0020/43319/E92823.pdf, accessed 8 February 2017).
42. Whitlock EP, Polen MR, Green CA, Orleans T, Klein J. Behavioral counseling interventions in primary care to reduce risky/harmful alcohol use by adults: a summary of the evidence for the U.S. Preventive Services Task Force. Ann Intern Med. 2004;140:557–68 (http://www.ncbi.nlm.nih.gov/pubmed/15068985, accessed 8 February 2017).
43. Johnson M, Jackson R, Guillaume L, Meier P, Goyder M. Barriers and facilitators to implementing screening and brief intervention for alcohol misuse: a systematic review of qualitative evidence. J Public Health. 2011;33(3):412–21 (http://jpubhealth.oxfordjournals.org/content/33/3/412.full, accessed 8 February 2017).
44. Blomberg R, Peck R, Moskowitz H, Burns M, Fiorentino D. Crash risk of alcohol involved driving: a case-control study. Stamford (CT): Dunlap and Associates, Inc; 2005 (http://www.dunlapandassociatesinc.com/crashriskofalcoholinvolveddriving.pdf, accessed 8 February 2017).
45. Ogden EJD, Moskowitz H. Effects of alcohol and other drugs on driver performance. Traffic Inj Prev. 2004;5:185–98 (http://www.ncbi.nlm.nih.gov/pubmed/15276919, accessed 8 February 2017).
46. Wagenaar AC, Toomey TL. Effects of minimum drinking age laws: review and analyses of the literature from 1960 to 2000. J Stud Alcohol Suppl. 2002;(14):206–25 (http://www.ncbi.nlm.nih.gov/pubmed/12022726, accessed 8 February 2017).
47. Hahn RA, Middleton JC, Elder R, Brewer R, Fielding J, Naimi TS et al. Effects of alcohol retail privatization on excessive alcohol consumption and related harms: a community guide systematic review. Am J Prev Med. 2012;42(4):418–27 (http://www.ncbi.nlm.nih.gov/pubmed/22424256, accessed 8 February 2017).
48. Chikritzhs T, Stockwell T. The impact of later trading hours for Australian public houses (hotels) on levels of violence. J Stud Alcohol. 2002;63:591–9 (http://www.ncbi.nlm.nih.gov/pubmed/12380856, accessed 8 February 2017).
49. Hahn RA, Kuzara JL, Elder R, Brewer R, Chattopadhyay S, Fielding J et al. Effectiveness of policies restricting hours of alcohol sales in preventing excessive alcohol consumption and related harms. Am J Prev Med. 2010;39(6):590–604 (https://www.ncbi.nlm.nih.gov/pubmed/21084080, accessed 8 February 2017).
50. Livingston M. Taxation and price control. In: Boyle P, Boffetta P, Lowenfels AB, Burns H, Brawley O, Zatonski W et al, editors. Alcohol: science, policy, and public health. Oxford: Oxford University Press; 2013 (https://global.oup.com/academic/product/alcohol-9780199655786?cc=dk&lang=en&, accessed 8 February 2017).
51. O'Mara RJ, Thombs DL, Wagenaar AC, Rossheim ME, Merves ML, Hou W et al. Alcohol price and intoxication in college bars. Alcohol Clin Ex Res. 2009;33(11):1973–80 (http://www.ncbi.nlm.nih.gov/pubmed/19719793, accessed 8 February 2017).
52. Bray JW, Loomis BR, Engelen M. You save money when you buy in bulk: does volume-based pricing cause people to buy more beer? Health Econ. 2009;18(5):607–18 (http://www.ncbi.nlm.nih.gov/pubmed/18770524, accessed 8 February 2017).
53. Jernigan DH. The global alcohol industry: an overview. Addiction. 2009;104(Suppl 1):6–12 (http://www.ncbi.nlm.nih.gov/pubmed/19133910, accessed 8 February 2017).
54. Wilkinson C, Room R. Warnings on alcohol containers and advertisements: international experience and evidence on effects. Drug Alcohol Rev. 2009;28(4):426–35 (http://www.ncbi.nlm.nih.gov/pubmed/19594797, accessed 8 February 2017).
55. Martineau F, Tyner E, Lorenc T, Petticrew M, Lock K. Population-level interventions to reduce alcohol-related harm: an overview of systematic reviews. Prev Med. 2013;57(4):278–96 (http://www.ncbi.nlm.nih.gov/pubmed/23811528, accessed 8 February 2017).
56. Fogarty J. The nature of the demand for alcohol: understanding elasticity. Br Food J Hyg Rev. 2006;108(4):316–32 (http://www.emeraldinsight.com/doi/abs/10.1108/00070700610657155, accessed 8 February 2017).
57. Wagenaar AC, Salois MJ, Komro KA. Effects of beverage alcohol price and tax levels on drinking: a meta-analysis of 1003 estimates from 112 studies. Addiction. 2009;104(2):179–90 (http://www.ncbi.nlm.nih.gov/pubmed/19149811, accessed 8 February 2017).

58. Gallet CA. The demand for alcohol: a meta-analysis of elasticities. Aust J Agric Resour Econ. 2007;51(2):121–35 (http://doi.wiley.com/10.1111/j.1467-8489.2007.00365.x, accessed 8 February 2017).
59. Mäkelä P, Osterberg E. Weakening of one more alcohol control pillar: a review of the effects of the alcohol tax cuts in Finland in 2004. Addiction. 2009;104(4):554–63 (https://www.ncbi.nlm.nih.gov/pubmed/19335654, accessed 8 February 2017).
60. Room R, Babor T, Rehm J. Alcohol and public health. Lancet. 2005;365(9458):519–30 (http://www.sciencedirect.com/science/article/pii/S0140673605178702, accessed 8 February 2017).
61. Gruenewald PJ, Ponicki WR, Holder HD, Romelsjö A. Alcohol prices, beverage quality, and the demand for alcohol: quality substitutions and price elasticities. Alcohol Clin Ex Res. 2006;30(1):96–105 (http://www.ncbi.nlm.nih.gov/pubmed/16433736, accessed 8 February 2017).
62. Meier P, Brennan A, Purshouse R, Taylor K, Rafia R, Booth A et al. Independent review of the effects of alcohol pricing and promotion: Part B. Modelling the potential impact of pricing and promotion policies for alcohol in England: results from the Sheffield Alcohol Policy Model. Sheffield: University of Sheffield; 2008 (http://www.shef.ac.uk/polopoly_fs/1.95621!/file/PartB.pdf, accessed 8 February 2017).
63. Anderson P, de Bruijn A, Angus K, Gordon R, Hastings G. Impact of alcohol advertising and media exposure on adolescent alcohol use: a systematic review of longitudinal studies. Alcohol Alcohol. 2009;44(3):229–43 (https://academic.oup.com/alcalc/article/44/3/229/178279/Impact-of-Alcohol-Advertising-and-Media-Exposure, accessed 8 February 2017).
64. Hollingworth W, Ebel BE, McCarty CA, Garrison MM, Christakis DA, Rivara FP. Prevention of deaths from harmful drinking in the United States: the potential effects of tax increases and advertising bans on young drinkers. J Stud Alcohol. 2006;67(2):300–8 (http://www.ncbi.nlm.nih.gov/pubmed/16562413, accessed 8 February 2017).
65. McCreanor T, Barnes HM, Kaiwai H, Borell S, Gregory A. Creating intoxigenic environments: marketing alcohol to young people in Aotearoa New Zealand. Soc Sci Med. 2008;67(6):938–46 (http://www.ncbi.nlm.nih.gov/pubmed/18619720, accessed 8 February 2017).
66. Room R, Osterberg E, Ramstedt M, Rehm J. Explaining change and stasis in alcohol consumption. Addict Res Theory. 2009;17(6):562–76 (http://www.tandfonline.com/doi/abs/10.3109/16066350802626966, accessed 8 February 2017).
67. Room R. Healthy is as healthy does: where will a voluntary code get us on international alcohol control? Addiction 2013;108(3):456–7 (http://onlinelibrary.wiley.com/doi/10.1111/j.1360-0443.2012.03980.x/full, accessed 8 February 2017).
68. Babor TF. Public health science and the global strategy on alcohol. Bull World Health Organ. 2010;88(9):643 (https://www.ncbi.nlm.nih.gov/pmc/articles/PMC2930373/, accessed 8 February 2017).
69. Thomas S, Paschall MJ, Grube JW, Cannon C, Treffers R. Underage alcohol policies across 50 California cities: an assessment of best practices. Subst Abuse Treat Prev Policy. 2012;7:26 (https://www.ncbi.nlm.nih.gov/pubmed/22734468, accessed 8 February 2017).
70. Treno AJ, Grube JW, Martin SE. Alcohol availability as a predictor of youth drinking and driving: a hierarchical analysis of survey and archival data. Alcohol Clin Exp Res. 2003;27/5):835–40 (http://www.ncbi.nlm.nih.gov/pubmed/12766629, accessed 8 February 2017).
71. Andrienko Y, Nemtsov A. Estimation of individual demand for alcohol. Moscow: Centre for Economic and Financial Research at New Economic School; 2006 (http://www.cefir.ru/papers/WP89_eng_Andrienko_Nemtsov.pdf, accessed 8 February 2017).
72. Burns H. Towards a global alcohol policy: current directions. In: Boyle P, Boffetta P, Lowenfels AB, Burns H, Brawley O, Zatonski W et al, editors. Alcohol: science, policy, and public health. Oxford: Oxford University Press; 2013 (https://global.oup.com/academic/product/alcohol-9780199655786?cc=dk&lang=en&, accessed 8 February 2017).
73. Status report on alcohol and health in 35 European countries 2013. Copenhagen: WHO Regional Office for Europe; 2013 (http://www.euro.who.int/__data/assets/pdf_file/0017/190430/Status-Report-on-Alcohol-and-Health-in-35-European-Countries.pdf?ua=1, accessed 8 February 2017).
74. European status report on alcohol and health 2010. Copenhagen: WHO Regional Office for Europe; 2010 (http://www.euro.who.int/__data/assets/pdf_file/0004/128065/e94533.pdf, accessed 8 February 2017).
75. Global status report on alcohol 2004. Geneva: World Health Organization; 2004 (http://www.who.int/substance_abuse/publications/global_status_report_2004_overview.pdf, accessed 8 February 2017).
76. Global status report on alcohol and health. Geneva: World Health Organization; 2011 (http://www.who.int/substance_abuse/publications/global_alcohol_report/msbgsruprofiles.pdf, accessed 8 February 2017).
77. Sands ES. Minimum-age laws and youth drinking: an introduction. In: Wechsler H, editor. Minimum drinking age laws. Lanham (MD): Lexington Books; 1980 (http://trid.trb.org/view.aspx?id=192466, accessed 8 February 2017).

78. Andreuccetti G, Carvalho HB, Cherpitel CJ, Yu Y, Ponce JC, Kahn T et al. Reducing the legal blood alcohol concentration limit for driving in developing countries: a time for change? Results and implications derived from a time-series analysis (2001-10) conducted in Brazil. Addiction. 2011;106(12):2124–31 (https://www.ncbi.nlm.nih.gov/pubmed/21631625, accessed 8 February 2017).
79. Eisenbach-Stangl I. Comparing European alcohol policies: what to compare? Vienna: European Centre for Social Welfare Policy and Research; 2011 (http://www.euro.centre.org/data/1308130015_25850.pdf, accessed 8 February 2017).
80. Maimon D, Browning CR. Underage drinking, alcohol sales and collective efficacy: informal control and opportunity in the study of alcohol use. Soc Sci Res. 2012;41(4):977–90 (http://www.ncbi.nlm.nih.gov/pubmed/23017864, accessed 8 February 2017).
81. Nordlund S. Policy norms, alcohol policy and drinking behaviour. In: Anderson P, Braddick F, Reynolds J, Gual A, editors. Alcohol policy in Europe: evidence from AMPHORA [e-book]. The AMPHORA project; 2012 (http://amphoraproject.net/w2box/data/e-book/AM_E-BOOK_2nd%20edition%20-%20final%20Sept%202013_c.pdf, accessed 8 February 2017).
82. Allamani A, Prina F. Why the decrease in consumption of alcoholic beverages in Italy between the 1970s and the 2000s – shedding light on an Italian mystery. Contemporary Drug Problems. 2007;34(2):187–98 (http://www.questia.com/library/journal/1P3-1465586971/why-the-decrease-in-consumption-of-alcoholic-beverages, accessed 8 February 2017).
83. Allamani A, Voller F, Decarli A, Casotto V, Pantzer K, Anderson P et al. Contextual determinants of alcohol consumption changes and preventive alcohol policies: a 12-country European study in progress. Subst Use Misuse. 2011;46(10):1288–303 (http://www.ncbi.nlm.nih.gov/pubmed/21692604, accessed 8 February 2017).
84. Joossens L, Raw M. The Tobacco Control Scale 2010 in Europe. Brussels: Association of the European Cancer Leagues; 2011 (http://www.krebshilfe.de/fileadmin/Inhalte/Downloads/PDFs/Kampagnen/TCS_2010_Europe.pdf, accessed 8 February 2017).
85. Draft action plan for the prevention and control of noncommunicable diseases 2013–2020. Report by the Secretariat. Geneva: World Health Organization; 2013 (EB132/7; http://apps.who.int/gb/ebwha/pdf_files/EB132/B132_7-en.pdf, accessed 8 February 2017).
86. McKee SA, Weinberger AH. How can we use our knowledge of alcohol-tobacco interactions to reduce alcohol use? Annu Rev Clin Psychol. 2013;9:649–74 (http://www.ncbi.nlm.nih.gov/pubmed/23157448, accessed 8 February 2017).
87. Cornelsen L, Normand C. Impact of the smoking ban on the volume of bar sales in Ireland: evidence from time series analysis. Health Econ. 2012;21(5):551–61 (http://www.ncbi.nlm.nih.gov/pubmed/22473645, accessed 8 February 2017).
88. Chisholm D, Doran C, Shibuya K, Rehm J. Comparative cost–effectiveness of policy instruments for reducing the global burden of alcohol, tobacco and illicit drug use. Drug Alcohol Rev. 2006;25(6):553–65 (http://www.ncbi.nlm.nih.gov/pubmed/17132573, accessed 8 February 2017).
89. Kypri K, O'Brien K, Miller P. Time for precautionary action on alcohol industry funding of sporting bodies. Addiction. 2009;104(12):1949–50 (http://onlinelibrary.wiley.com/doi/10.1111/j.1360-0443.2009.02711.x/full, accessed 8 February 2017).

ANNEX 1. LIST OF SURVEY QUESTIONS USED FOR THE EAPA COMPOSITE INDICATORS ARRANGED BY SIS

1. Leadership, awareness and commitment

1.1. National policy on alcohol
Is there a written national policy on alcohol specific to your country? *A written national policy on alcohol is an organized set of values, principles and objectives for reducing the burden attributable to alcohol in a population which is adopted at the national level.*

- ☐ National policy
- ☐ Subnational: description of subnational policy/regional variations:
- ☐ No

Is the written national policy on alcohol multisectoral?

☐ No ☐ Yes

For the implementation of the written national policy on alcohol, is there a national action plan?

☐ No ☐ Yes

Is there currently a process of developing a written national policy on alcohol or of revising the adopted one? *Check (✓) one only.*

- ☐ No
- ☐ Yes, revising the adopted one
- ☐ Yes, developing a written national policy on alcohol

1.2. Definition of alcoholic beverage
In your country, is there a standard legal definition of an alcoholic beverage that is used by your government?

☐ No ☐ Yes

If YES, what is the standard legal definition of an alcoholic beverage in your country? *Please include the % alcohol by volume if applicable, e.g. "All types of beverages over 0.5% alcohol by volume".*

1.3. Definition of standard drink
In your country, is there a definition of a standard drink used at the national level?

☐ No ☐ Yes

If YES, how much is a standard drink in grams of pure alcohol?

1.4. Awareness activities

In the last three years, did you have any nationwide awareness-raising activities?

☐ No ☐ Yes. *Please specify. Check (✓) all that apply.*

☐ Young people's drinking
☐ Drink–driving
☐ For indigenous peoples
☐ Impact of alcohol on health
☐ Social harms (harms to others than the drinker)
☐ Other, please specify
☐ Illegal/surrogate alcohol
☐ Alcohol and pregnancy
☐ Alcohol at work

In your country, which of the following tools/programmes are used for prevention of substance use and substance use disorders? *Please answer for alcohol use and alcohol use disorders. Please precise the estimated level of coverage (%) of the target population.*

☐ There are no tools/programmes

	Mass media (audiovisual)	Mass media (print)	Advertisements in public places (posters)
None (0%)	☐	☐	☐
Some (1–30%)	☐	☐	☐
High (31–60%)c	☐	☐	☐
Very high (61–100%)	☐	☐	☐

2. Health services' response

2.1. Screening and brief interventions for harmful and hazardous alcohol use

In your country are there clinical guidelines for brief interventions that have been approved or endorsed by at least one health care professional body?

☐ No ☐ Yes

What is the proportion of primary health care services that have implemented *screening* and brief interventions for harmful and hazardous substance use at the national level? *Specify for alcohol use. Screening can be simply by asking about substance use and not necessarily involving standardized screening questionnaires or testing.*

	Routine screening (for majority of patients)	Selective screening (for minority of patients)
None (0)	☐	☐
Few (1-10%)	☐	☐
Some (11–30%)	☐	☐
Many (31–60%)	☐	☐
Most (61–100%)	☐	☐
Unknown	☐	☐

What is the proportion of ante-natal services that have implemented screening and brief interventions for harmful and hazardous substance use at the national level? *Specify for alcohol use. Screening can be simply by asking about substance use and not necessarily involving standardized screening questionnaires or testing.*

None (0)	☐
Few (1-10%)	☐
Some (11–30%)	☐
Many (31–60%)	☐
Most (61–100%)	☐
Unknown	☐

2.2. Special treatment programmes

In your country, are there special treatment programmes for women as well as for children and adolescents with substance use disorders? *Please specify for alcohol use disorders and in which area of the country they are located. Please tick all that apply.*

	Special treatment programmes for women	Special treatment programmes for children and adolescents
No	☐	☐
Yes, in the capital city	☐	☐
Yes, in other major cities[a]	☐	☐
Yes, in other areas[b]	☐	☐

[a] Major cities refers to cities with relatively large population and available tertiary and higher levels of health care that includes highly specialized facilities such
as university hospitals or highly specialized treatment centres such as for neurosurgery or radiology.

[b] Other areas refers to urban and rural areas outside the capital and major cities.

2.3. Pharmacological treatment

In your country, which of the following medications are available? *Specify if it is registered, available in publicly funded treatment services and if the dosing is supervised. Check (✓) all that apply*

Medication	Formulation	For the treatment of:	Registered in the country		Is it available for use in publicly funded treatment services for this indication?		Is outpatient dosing generally supervised?[a]	
			Yes	No	Yes	No	Yes	No
Acamprosate	Tablets	alcohol dependence	☐	☐	☐	☐	NA	
Buprenorphine	Sublingual tablets	opioid dependence	☐	☐	☐	☐	☐	☐
Buprenorphine/ naloxone	Sublingual tablets	opioid dependence	☐	☐	☐	☐	☐	☐
Buprenorphine/ naloxone	Sublingual film	opioid dependence	☐	☐	☐	☐	☐	☐
Diazepam (or other long acting benzodiazepines)	Tablets	alcohol withdrawal	☐	☐	☐	☐	☐	☐
Diazepam (or other long acting benzodiazepines)	Tablets	benzodiazepine withdrawal	☐	☐	☐	☐	☐	☐
Clonidine	Tablets	opioid withdrawal	☐	☐	☐	☐	NA	
Disulfiram	Tablets	alcohol dependence	☐	☐	☐	☐	☐	☐
Lofexidine	Tablets	opioid withdrawal	☐	☐	☐	☐	NA	
Methadone	Liquid	opioid dependence	☐	☐	☐	☐	☐	☐
Methadone	Tablets	opioid dependence	☐	☐	☐	☐	☐	☐
Naloxone	For injection	opioid overdose	☐	☐	☐	☐	NA	
Naltrexone	Tablets	alcohol dependence	☐	☐	☐	☐	NA	
Naltrexone	Tablets	opioid dependence	☐	☐	☐	☐	☐	☐

[a] Supervision for methadone, buprenorphine, diazepam, disulfiram and naltrexone dosing for outpatients: tick YES if outpatients are required to have doses supervised daily unless an individual assessment determined that daily supervision of dosing is not necessary. In supervised methadone treatment, for example, patients come each day for their dose at the beginning of treatment until they are assessed as suitable to receive take-home methadone.

3. Community action

3.1. School-based prevention and reduction of alcohol-related harm

In your country, do you have national guidelines for the prevention and reduction of alcohol-related harm in school settings?

☐ No ☐ Yes

In your country, is there a legal obligation for schools to carry out alcohol (or broader alcohol and other substance use) prevention as part of the school curriculum or as part of school health policies?

☐ No ☐ Yes

3.2. Workplace-based alcohol problem prevention and counselling
In your country, are there any national guidelines for alcohol problem prevention and counselling at workplaces?

☐ No ☐ Yes

In your country, is there legislation on alcohol testing at workplaces?

☐ No ☐ Yes

In your country, are workplace programmes used for the prevention of substance use and substance use disorders? *Please answer for alcohol use and alcohol use disorders. Please precise the estimated level of coverage (%) of the target population.*

☐ There are no tools/programmes

None (0%)	☐
Some (1–30%)	☐
High (31–60%)c	☐
Very high (61–100%)	☐

3.3. Community-based interventions to reduce alcohol-related harm
In your country, are there national guidelines for implementing effective community-based interventions to reduce alcohol-related harm?

☐ No ☐ Yes

In your country, are there any communitybased interventions/projects involving stakeholders (nongovernmental organizations, economic operators, others)?

☐ No ☐ Yes. *Please specify the most important sectors involved. Check (✓) all that apply.*

☐ Nongovernmental organizations
☐ Economic operators
☐ Local government bodies
☐ Others. *Please specify:*

In your country, are there community-based programmes used for prevention of substance use and substance use disorders? *Please answer for alcohol use and alcohol use disorders. Please precise the estimated level of coverage (%) of the target population.*

☐ There are no tools/programmes

None (0%)	☐
Some (1–30%)	☐
High (31–60%)c	☐
Very high (61–100%)	☐

4. Drink–driving policies and countermeasures

4.1. Maximum legal blood alcohol concentration (BAC) limit when driving a vehicle
At the national level, what is the maximum legal BAC when driving a vehicle, for each of the following groups? (e.g., 0.05%; usually, from 0% to 0.10%). *Enter the BAC in % or "None" if there is no maximum legal BAC.*

General population: 0._ _ %
Young/novice drivers: 0._ _ %

4.2. Enforcement using sobriety checkpoints
Do you have sobriety checkpoints? *Sobriety checkpoints are checkpoints or roadblocks established by the police on public roadways to control for drink–driving.*

☐ Yes ☐ No

4.3. Enforcement using random breath-testing
Do you have random breath testing? *Random breath testing means that any driver can be stopped by the police at any time to test the breath for alcohol consumption*.

☐ Yes ☐ No

4.4. Penalties
What are the penalties for drink–driving in your country? *Check (✓) all that apply.*

☐ Fines
☐ Penalty points
☐ Short-term detention
☐ Vehicle impounded
☐ Mandatory treatment
☐ Mandatory education and counselling
☐ Driving licence suspension
☐ Driving licence revoked
☐ Imprisonment
☐ Community/public service
☐ Ignition interlock
☐ None

5. Availability of alcohol

5.1. Lowest age limit for on-premise alcohol service and off-premise alcohol sale
What are the legal age limits at the national level, for the following? *Enter age limit (in years) or "None" if there is no age limit. Legal age limit means that alcoholic beverages cannot be served/sold to a person under this age.*

	Beer	Wine	Spirits
On-premise sales (serving) (cafe, pub, bar, restaurant)	___ years	___ years	___ years
Off-premise sales (selling) (take-away from, for example, shop, supermarket)	___ years	___ years	___ years

5.2. Control of retail sales
If the control for production and sale of alcohol is at the national level, do you have government monopoly? *Please check (✓) the appropriate answer(s). Government monopoly means full or almost complete government control.*

	Beer	Wine	Spirits
Monopoly on production	☐ Yes ☐ No	☐ Yes ☐ No	☐ Yes ☐ No
Monopoly on retail sales	☐ Yes ☐ No	☐ Yes ☐ No	☐ Yes ☐ No

If the control for production and sale of alcohol is at the national level, do you have licensing? *Please check (✓) the appropriate answer(s). Licensing means partial government control where a license is required.*

	Beer	Wine	Spirits
Licence for production	☐ Yes ☐ No	☐ Yes ☐ No	☐ Yes ☐ No
Licence for retail sales	☐ Yes ☐ No	☐ Yes ☐ No	☐ Yes ☐ No

5.3. Restrictions on alcohol availability by time

Please provide information on existing restrictions for the on-premise sales of beer, wine and spirits at the national level. *Check (✓) the appropriate answers. On-premise sales means serving in, for example, a cafe, pub, bar, restaurant.*

	Beer	Wine	Spirits
Hours of sales	☐ Yes ☐ No	☐ Yes ☐ No	☐ Yes ☐ No
Days of sales	☐ Yes ☐ No	☐ Yes ☐ No	☐ Yes ☐ No

Please provide information on existing restrictions for the off-premise sales of beer, wine and spirits at the national level. *Check (✓) the appropriate answers. Off-premise sales means selling as take-away in, for example, a shop or supermarket.*

	Beer	Wine	Spirits
Hours of sales	☐ Yes ☐ No	☐ Yes ☐ No	☐ Yes ☐ No
Days of sales	☐ Yes ☐ No	☐ Yes ☐ No	☐ Yes ☐ No

5.4. Restrictions on alcohol availability by place

Please provide information on existing restrictions for the on-premise sales of beer, wine and spirits at the national level. *Check (✓) the appropriate answers. On-premise sales means serving in, for example, a cafe, pub, bar, restaurant.*

	Beer	Wine	Spirits
Locations of sales	☐ Yes ☐ No	☐ Yes ☐ No	☐ Yes ☐ No
Density of outlets	☐ Yes ☐ No	☐ Yes ☐ No	☐ Yes ☐ No

Please provide information on existing restrictions for the off-premise sales of beer, wine and spirits at the national level. *Check (✓) the appropriate answers. Off-premise sales means selling as take-away in, for example, a shop or supermarket.*

	Beer	Wine	Spirits
Locations of sales	☐ Yes ☐ No	☐ Yes ☐ No	☐ Yes ☐ No
Density of outlets	☐ Yes ☐ No	☐ Yes ☐ No	☐ Yes ☐ No

5.5. Restrictions on sales at specific events

Please provide information on existing restrictions for the on-premise sales of beer, wine and spirits at the national level. *Check (✓) the appropriate answers. On-premise sales means serving in, for example, a cafe, pub, bar, restaurant.*

	Beer	Wine	Spirits
Sales at specific events e.g., football games)	☐ Yes ☐ No	☐ Yes ☐ No	☐ Yes ☐ No

Please provide information on existing restrictions for the off-premise sales of beer, wine and spirits at the national level. *Check (✓) the appropriate answers. Off-premise sales means selling as take-away in, for example, a shop or supermarket.*

	Beer	Wine	Spirits
Sales at specific events e.g., football games)	☐ Yes ☐ No	☐ Yes ☐ No	☐ Yes ☐ No

5.6. Alcohol-free public environments

Please provide information on the extent to which different public environments are alcohol-free in your country. *Check (✓) the appropriate column. Partial statutory restriction means that certain alcoholic beverages are forbidden or some offices/ buildings/places are alcohol-free. Voluntary agreement/self-regulation means that local governments and municipalities have their own regulations or the alcoholic beverage industry follows its internal voluntary rules.*

restriction	Ban	Partial statutory restriction	Voluntary/ self-regulated	No
Educational buildings	☐	☐	☐	☐
Public transport	☐	☐	☐	☐
Parks, streets	☐	☐	☐	☐
Sporting events	☐	☐	☐	☐

6. Marketing of alcoholic beverages

6.1. Legally binding restrictions on alcohol advertising

Are there legally binding restrictions on alcohol advertising at the national level?

☐ No ☐ Yes

If YES, please specify the restrictions on alcohol advertising. Use letters to indicate the type of beverage (B=BEER), (W=WINE) and (S=SPIRITS) for which there are restrictions. *Partial statutory restriction means that the restriction applies during a certain time of day or for a certain place, or to the content of events, programmes, magazines, films and so on. Voluntary agreement means that the alcoholic beverage industry follows its internal voluntary rules.*

	Ban	Partial statutory restriction: Time/place	Partial statutory restriction: content	Voluntary/ self-regulated	No restriction
Public service/national TV					
Commercial/private TV					
National radio					
Local radio					
Print media (newspapers etc.)					
Billboards					
Points of sale					
Cinema					
Internet					
Social media (Facebook etc.)					

6.2. Legally binding restrictions on product placement
Are there legally binding restrictions on alcohol product placement at the national level? *Product placement means that economic operators sponsor TV or film productions if their product is shown in these productions.*

☐ No ☐ Yes

If YES, please specify the restrictions on product placement. Use letters to indicate the type of beverage (B=BEER), (W=WINE) and (S=SPIRITS) for which there are restrictions. *Partial statutory restriction means that the restriction applies during a certain time of day or for a certain place, or to the content of events, programmes, magazines, films and so on. Voluntary agreement means that the alcoholic beverage industry follows its internal voluntary rules.*

	Ban	Partial statutory restriction: Time/place	Partial statutory restriction: content	Voluntary/ self-regulated	No restriction
Public service/national TV					
Commercial/private TV					
Films/movies					

6.3. Legally binding restrictions on industry sponsorship for sporting and youth events
Are there legally binding restrictions on alcoholic beverage industry sponsorship at the national level?

☐ No ☐ Yes

If YES, please specify the restrictions on industry sponsorship. Use letters to indicate the type of beverage (B=BEER), (W=WINE) and (S=SPIRITS) for which there are restrictions. *Partial statutory restriction means that the restriction applies during a certain time of day or to some events, programmes, magazines, films and so on. Voluntary agreement/ self-regulation means that the alcoholic beverage industry follows its internal voluntary rules*.

	Ban	Partial statutory restriction	Voluntary/ self-regulated	No restriction
Industry sponsorship of sporting events				
Industry sponsorship of youth events such as concerts				

6.4. Legally binding restrictions on sales promotions by producers, retailers and owners of pubs and bars
Are there legally binding restrictions on sales promotion from producers, retailers (including supermarkets) and owners of pubs and bars at the national level?

☐ No ☐ Yes

If YES, please specify the restrictions on sales promotion. Use letters to indicate the type of beverage (B=BEER), (W=WINE) and (S=SPIRITS) for which there are restrictions. *Partial statutory restriction means that the restriction applies during a certain time of day or to some events, programmes, magazines, films and so on. Voluntary agreement/ self-regulation means that the alcoholic beverage industry follows its internal voluntary rules.*

	Ban	Partial statutory restriction	Voluntary/ self-regulated	No restriction
Sales promotion from producers (for example, parties and events)				
Below costs sales promotions from retailers (including supermarkets)				
Free drinks sales promotions from owners of pubs and bars				

7. Pricing policies

7.1. Adjustment of taxation level for inflation
Is the level of taxation (excise tax or special tax on alcohol other than excise tax) for alcoholic beverages adjusted for inflation in your country? *Please specify how often the level of taxation is adjusted for inflation (e.g. every 3 months/ every year):*

Beer	☐ No ☐ Yes ⟶	every |__|__| months/every |__|__| years
Wine	☐ No ☐ Yes ⟶	every |__|__| months/every |__|__| years
Spirits	☐ No ☐ Yes ⟶	every |__|__| months/every |__|__| years
Other (most popular country-specific alcoholic beverage); please specify name: ______________________	☐ No ☐ Yes ⟶	every |__|__| months/every |__|__| years
% alcohol by	volume: _ _ % and:	

7.2. Affordability of alcoholic beverages

Please specify the average retail price for alcoholic beverages.

	Quantity in cL	Reference brand *(market leader)*	Average retail price *(in local currency)*
Beer: most popular brand of beer			
Wine: table wine/ordinary wine			
Spirits: most popular local brand			
Spirits: most popular imported brand			
Other (most popular country-specific alcoholic beverage); please specify % alcohol by volume: _ _ % and name:			

7.3. Other price measures

Do you have any price measures other than taxation in your country? *Price measures other than taxation means, for example regulation of the price of non-alcoholic and alcoholic beverages, such as making a non-alcoholic beverage cheaper than an alcoholic beverage.*

☐ No ☐ Yes. *Please specify. Check (✓) all that apply.*

- ☐ Minimum price policy
- ☐ Requirement to offer non-alcoholic beverages at a lower price
- ☐ Additional levy on specific products (for example, on alcopops), please specify:
- ☐ Price measures to discourage underage drinking or high-volume drinking. *Please specify:*
- ☐ Ban on below-cost selling
- ☐ Ban on volume discounts
- ☐ Other, please specify:

8. Reducing the negative consequences of drinking and alcohol intoxications

8.1. Server training

In your country, is there any systematic alcohol server training (for servers of pubs, bars, restaurants) on a regular basis? *Check (✓) all that apply. Server training means a form of occupational training provided to people serving alcohol such as bar and restaurant staff, waiting staff or people serving at catered events. Alcohol server training promotes the safe service of alcoholic beverages to customers (such as not serving to intoxication, not serving to those already intoxicated or to minors). Alcohol server training can be regulated and mandated by state or local laws.*

- ☐ No
- ☐ Yes, organized by enforcement agencies
- ☐ Yes, organized by the private sector
- ☐ Yes, organized by other, please specify:

8.2. Health warning labels

Are health warning labels legally required on alcohol advertisements in your country at the national level?

☐ No ☐ Yes

Are health warning labels legally required on the containers/bottles of alcoholic beverages in your country at the national level?

☐ No ☐ Yes

9. Reducing the public health impact of illicit alcohol and informally produced alcohol

9.1. Use of duty paid or excise stamps on alcohol containers
Do you use duty-paid, excise or tax stamps or labels on alcoholic beverage containers/bottles in your country?

Beer	☐ No	☐ Yes
Wine	☐ No	☐ Yes
Spirits	☐ No	☐ Yes

9.2. Estimates of unrecorded alcohol consumption
What are the main components of the national system of monitoring alcohol consumption? *Check (✓) all that apply.*

☐ Regular estimation of consumption of unrecorded (informally/illegally produced) alcohol based on expert opinion
☐ Regular estimation of consumption of unrecorded (informally/illegally produced) alcohol based on research focused on unrecorded alcohol consumption
☐ Regular estimation of consumption of unrecorded (informally/illegally produced) alcohol based on indirect estimates using government data on confiscated/seized alcohol
☐ Regular estimation of consumption of unrecorded (informally/illegally produced) alcohol based on indirect estimates using survey data
☐ Regular estimation of consumption of unrecorded (informally/illegally produced) alcohol based on indirect estimates using other data. *Please specify other data for estimation of unrecorded:*

9.3. Legislation to prevent illegal production and sale of alcoholic beverages
Do you have any national legislation in your country to prevent illegal production and/or sale of home- or informally produced alcoholic beverages?

☐ No
☐ Yes, to prevent illegal production
☐ Yes, to prevent illegal sale

10. Monitoring and surveillance

10.1. National monitoring system

In your country, do you have a national system for monitoring alcohol consumption, its health and social consequences? *Check (✓) all that apply. A national system for monitoring alcohol consumption, its health and social consequences refers to a data repository including a range of population-based and health facility data. The main population-based sources of health information are censuses, household surveys and (sample) vital registration systems. The main health facility-related data sources are public health surveillance, health services data and health system monitoring data.*

☐ Yes, with data collected on alcohol consumption
☐ Yes, with data collected on health consequences of alcohol consumption
☐ Yes, with data collected on social consequences of alcohol consumption
☐ Yes, with data collected on alcohol policy responses
☐ No

What are the main components of the national system of monitoring alcohol consumption? *Check (✓) all that apply.*

☐ Sales data for alcoholic beverages
☐ National population-based surveys including questions on alcohol consumption. *Please specify: (i) how often these types of survey are implemented (e.g. every 3 years): every _ _ years; and (ii) the last year of survey implementation (e.g. year 2011):*

Are there regular reports available?

☐ Yes. *Please specify/indicate the year of last publication/release and web link or reference.*
Year:
Web link or reference:
☐ No

What resources are secured for the national monitoring system?

☐ Institution/organization/department with the mandated function of a national monitoring centre. *Please provide the name and location of the institution/organization/department with such a monitoring function:*

☐ A person with the mandated function of monitoring the situation on alcohol and health.

10.2. National surveys
What are the main components of the national system of monitoring alcohol consumption?

☐ National youth (including school-based) surveys including questions on alcohol consumption. *Please specify: (i) how often these types of surveys are implemented (every _ _ years); and (ii) the last year of survey implementation (_ _ _ _):*

Do you have national surveys on the rates of heavy episodic drinking (binge drinking)* among adults (15+ years)? *The definition of heavy episodic drinking/binge drinking here should be 60+ g of pure alcohol on at least one occasion weekly during the past 12 months.*

☐ Yes ☐ No

ANNEX 2. DETAILED SCORING RUBRICS FOR THE EAPA COMPOSITE INDICATORS

**1.**	**Leadership, awareness and commitment (maximum 23 points (p.))**				
**1.1**	**National policy on alcohol**<br>*An adopted written national policy on alcohol is defined as a written organized set of values, principles and objectives for reducing the burden attributable to alcohol in a population.*				
	Written national policy on alcohol	☐ Adopted (2 p.)	☐ In development (1 p.)		☐ No (0 p.)
	Written national policy on alcohol is multisectoral	☐ Yes (1 p.)	☐ N/A (0 p.)		☐ No (0 p.)
	Written national policy on alcohol policy is accompanied by a national action plan for implementation	☐ Yes (1 p.)	☐ N/A (0 p.)		☐ No (0 p.)
	Multiplier x 3				
**1.2**	**Definition of alcoholic beverage**<br>*A beverage over a certain percentage of alcohol by volume is defined as an alcoholic beverage.*				
	An alcoholic beverage is legally defined as a beverage over 0.1–2.8% alcohol by volume	☐ Yes (1 p.)			☐ No (0 p.)
	Multiplier x 2				
**1.3**	**Definition of standard drink**<br>*A definition of a standard drink (in grams of pure alcohol) is used at the national level.*				
	A standard drink is defined as 8–12 g of pure alcohol	☐ Yes (1 p.)			☐ No (0 p.)
	Multiplier x 1				
**1.4**	**Awareness activities**<br>*Awareness activities are provided pertaining to the following topics: young people's drinking, drink–driving, indigenous peoples, impact on health, social harms, illegal/surrogate alcohol, alcohol at work, or pregnancy and alcohol.*				
	Implementation of national awareness activities within last three years	☐ 6 or more topics (3 p.)	☐ 4–5 topics (2 p.)	☐ 1–3 topics (1 p.)	☐ None (0 p.)
	Tools/programmes used for the prevention of alcohol use and alcohol use disorders (audiovisual mass media, print mass media or advertisements in public places) cover at least 31% of the target population	☐ Yes (1 p.)			☐ No (0 p.)
	Multiplier x 2				

2.	Health services' response					
**2.1**	**Screening and brief interventions for harmful and hazardous alcohol use**<br>*Screening and short-term interventions are implemented for harmful and hazardous alcohol use. Screening can consist of simple questions about alcohol use and does not necessarily involve standardized screening questionnaires or testing.*					
	Clinical guidelines for brief interventions on alcohol	☐ Yes (2 p.)				☐ No (0 p.)
	Proportion of primary health care services that have implemented routine (for a majority of patients) and/or selective (for a minority of patients) screening and brief intervention	☐ Most (61–100%) (4 p.)	☐ Many (31–60%) (3 p.)	☐ Some (11–30%) (2 p.)	☐ Few (1–10%) (1 p.)	☐ None (0 p.)
	Proportion of antenatal services that have implemented screening and brief interventions for harmful and hazardous alcohol use at the national level	☐ Most (61–100%) (4 p.)	☐ Many (31–60%) (3 p.)	☐ Some (11–30%) (2 p.)	☐ Few (1–10%) (1 p.)	☐ None (0 p.)
	Multiplier x 3					
**2.2**	**Special treatment programmes**					
	Special treatment programmes for women with alcohol use disorders are available in major cities or other areas	☐ Yes (2 p.)				☐ No (0 p.)
	Special treatment programmes for children and adolescents with alcohol use disorders are available in major cities or other areas	☐ Yes (2 p.)				☐ No (0 p.)
	Multiplier x 2					
**2.3**	**Pharmacological treatment**<br>*Medications are available for the treatment of alcohol dependence or alcohol withdrawal.*					
	The following medications are available for the treatment of alcohol dependence or alcohol withdrawal:<br>☐ Acamprosate (1 p.)<br>☐ Diazepam (or other long-acting benzodiazepines) (1 p.)<br>☐ Disulfiram (1 p.)<br>☐ Naltrexone (1 p.)					☐ No (0 p.)
	Multiplier x 3					

3.	Community action (maximum 34 p.)		
**3.1**	**School-based prevention and reduction of alcohol-related harm**		
	National guidelines are available for the prevention and reduction of alcohol-related harm in school settings	☐ Yes (2 p.)	☐ No (0 p.)
	Schools are legally obliged to carry out alcohol (or broader alcohol and other substance use) prevention as part of the school curriculum or as part of school health policies	☐ Yes (2 p.)	☐ No (0 p.)
	Multiplier x 2		
**3.2**	**Workplace-based alcohol problem prevention and counselling**		
	National guidelines are available for prevention and counselling for alcohol problems at workplaces	☐ Yes (2 p.)	☐ No (0 p.)
	Legislation is in place on alcohol testing at workplaces	☐ Yes (1 p.)	☐ No (0 p.)
	Workplace programmes for the prevention of alcohol use and alcohol use disorders cover at least 31% of the target population	☐ Yes (3 p.)	☐ No (0 p.)
	Multiplier x 2		
**3.3**	**Community-based interventions to reduce alcohol-related harm**		
	National guidelines are available for implementing effective community-based interventions to reduce alcohol-related harm	☐ Yes (2 p.)	☐ No (0 p.)
	External stakeholders are involved in community-based interventions and projects	☐ Yes (2 p.)	☐ No (0 p.)
	Community-based programmes for the prevention of alcohol use and alcohol use disorders cover at least 31% of the target population	☐ Yes (3 p.)	☐ No (0 p.)
	Multiplier x 2		

4.	Drink–driving policies and countermeasures (maximum 66 p.)			
**4.1**	**Maximum legal blood alcohol concentration (BAC) limit when driving a vehicle**<br>*The legal maximum BAC (measured as mass per volume) allowed while driving a vehicle in a country.*			
	General BAC limit	☐ ≤0.02% (3 p.)	☐ >0.02% but ≤0.05% (2 p.)	☐ >0.05% (0 p.)
	BAC for young/novice drivers	☐ ≤0.02% (2 p.)	☐ >0.02% but ≤0.05% (1 p.)	☐ >0.05% (0 p.)
	Multiplier x 5			
**4.2**	**Enforcement using sobriety checkpoints**<br>*Police checkpoints are used to enforce alcohol laws. Sobriety checkpoints are checkpoints or roadblocks established by the police on public roadways to control for drink–driving.*			
	Sobriety checkpoints are used	☐ Yes (3 p.)		☐ No (0 p.)
	Multiplier x 3			
**4.3**	**Enforcement using random breath-testing**<br>*Random breath-testing is used to enforce alcohol laws. Random breath-testing is defined as a test given by the police to drivers chosen by chance. It means that any driver can be stopped by the police at any time to test the breath for alcohol consumption.*			
	Random breath-testing is used	☐ Yes (4 p.)		☐ No (0 p.)
	Multiplier x 4			
**4.4**	**Penalties**<br>*Penalties include: community/public service, short-term detention, fines, penalty points, licence suspension, licence revocation, imprisonment, impounding of vehicle, ignition interlocks (alcolocks), mandatory treatment, and mandatory education and counselling imposed on drivers for disregarding drink–driving laws.*			
	Penalties	☐ At least 4 different types of penalty implemented (4 p.)	☐ 1–3 different types of penalty implemented (2 p.)	☐ None (0 p.)
	Multiplier x 4			

5.	Availability of alcohol (maximum 94 p.)				
**5.1**	**Lowest age limit for on-premise alcohol service and off-premise alcohol sale**<br>*These are the lowest ages at which a person can be served alcoholic beverages on premises in a country (alcoholic beverages cannot be served to a person under this age) and sold alcoholic beverages for consumption off the premises in a country (alcoholic beverages cannot be sold to a person under this age).*				
	☐ ≥20 years (4 p.)		☐ 18–19 years (3 p.)		☐ <18 years (0 p.)
	Multiplier x 4				
**5.2**	**Control of retail sales**<br>*Licensing refers to partial government control of the sale of alcoholic beverages. A monopoly refers to a government monopoly (full control) of the sale of alcoholic beverages.*				
	☐ Full monopoly (beer and wine and spirits) (4 p.)	☐ Partial monopoly (beer or wine or spirits) (3 p.)	☐ Full licensing (beer and wine and spirits) (2 p.)	☐ Partial licensing (beer or wine or spirits) (1 p.)	☐ None (0 p.)
	Multiplier x 3				
**5.3**	**Restrictions on alcohol availability by time**<br>*There are regulated limits on the time (hours/days) of sales of alcoholic beverages.*				
	☐ Comprehensive restriction on either days or hours of sales (beer and wine and spirits) for **both** on-premises and off-premises sales (4 p.)	☐ Comprehensive restriction on either days or hours of sales (beer and wine and spirits) for **either** on-premises or off-premises sales (3 p.)	☐ Partial restriction on either days or hours of sales (beer or wine or spirits) for **both** on-premises and off-premises sales (2 p.)	☐ Partial restriction on either days or hours of sales (beer or wine or spirits) for **either** on-premises or off-premises sales (1 p.)	☐ None (0 p.)
	Multiplier x 3				
**5.4**	**Restrictions on alcohol availability by place**<br>*There are regulated limits on the location (places/density) of sales of alcoholic beverages.*				
	☐ Comprehensive restriction on either location or density of sales (beer and wine and spirits) for **both** on-premises and off-premises sales (4 p.)	☐ Comprehensive restriction on either location or density of sales (beer and wine and spirits) for **either** on-premises or off-premises sales (3 p.)	☐ Partial restriction on either location or density of sales (beer or wine or spirits) for **both** on-premises and off-premises sales (2 p.)	☐ Partial restriction on either location or density of sales (beer or wine or spirits) for **either** on-premises or off-premises sales (1 p.)	☐ None (0 p.)
	Multiplier x 3				

5.5 Restrictions on sales at specific events
There are regulated limits on the sales of alcoholic beverages during specific events (such as football games).

☐ Comprehensive restrictions (beer and wine and spirits) (3 p.)	☐ Partial restrictions (beer or wine or wine or spirits) (2 p.)	☐ None (0 p.)

Multiplier x 3

5.6 Alcohol-free public environments
Alcohol use is restricted in public places such as public transport, parks and streets, educational buildings and sporting events.

Restriction on alcohol consumption on public transport	☐ Partial restriction or ban (2 p.)	☐ None or voluntary agreement/ self-regulation (0 p.)
Restriction on alcohol consumption in public areas (such as parks or streets)	☐ Partial restriction or ban (3 p.)	☐ None or voluntary agreement/ self-regulation (0 p.)
Restriction on alcohol consumption in educational buildings	☐ Partial restriction or ban (3 p.)	☐ None or voluntary agreement/ self-regulation (0 p.)
Restriction alcohol consumption at sporting events	☐ Partial restriction or ban (3 p.)	☐ None or voluntary agreement/ self-regulation (0 p.)

Multiplier x 3

6. Marketing of alcoholic beverages (see also rubric 6a) (maximum 48 p.)

6.1 Legally binding restrictions on alcohol advertising
Alcohol advertising is defined as the promotion of alcoholic beverages by the alcohol industry through a variety of media: national television, cable television, national radio, local radio, print media, cinemas, billboards, points of sale, internet and social media. The level of restriction may be a total ban, partial statutory restriction or voluntary agreement/self-regulation. (Partial statutory restriction means that the restriction applies during a certain time of day or to some events, programmes, magazines, films or suchlike. Voluntary agreement/self-regulation means that the alcoholic beverage industry follows its internal voluntary rules.)

☐ Total ban (3 p.)	☐ Partial statutory restriction (2 p.)	☐ Voluntary agreement/ self-regulation (1 p.)	☐ None (0 p.)

Multiplier see rubric 6a

6.2 Legally binding restrictions on product placement
Product placement refers to the sponsorship of, for example, television productions by economic operators if their alcoholic beverage is shown in these productions. Media include: public service/national television, commercial/private television and films. The level of restriction may be a total ban, partial statutory restriction or voluntary agreement/self-regulation.

☐ Total ban (3 p.)	☐ Partial statutory restriction (2 p.)	☐ Voluntary agreement/ self-regulation (1 p.)	☐ None (0 p.)

Multiplier see rubric 6a

6.3 Legally binding restrictions on industry sponsorship for sporting and youth events
Sponsorship refers to the support of an event financially or through the provision of products or services as part of brand identification and marketing.

☐ Total ban (3 p.)	☐ Partial statutory restriction (2 p.)	☐ Voluntary agreement/ self-regulation (1 p.)	☐ None (0 p.)

Multiplier see rubric 6a

6.4 Legally binding restrictions on sales promotions by producers, retailers and owners of pubs and bars
Restrictions are legally enforced on the promotion of alcohol sales in a country by, for example, producers (parties and events), retailers (including supermarkets) in the form of sales below cost (for example, two for the price of one, happy hours), or owners of pubs and bars (serving alcohol-free products). Sales promotion refers to marketing practices designed to facilitate the purchase of a product.

☐ Total ban (3 p.)	☐ Partial statutory restriction (2 p.)	☐ Voluntary agreement/ self-regulation (1 p.)	☐ None (0 p.)

Multiplier see rubric 6a

6a. Marketing of alcoholic beverages

A nested banding approach is employed. Points are awarded for multiple items (such as various advertising platforms) based on the level of restriction applied to different types of beverage. The sum of points across the items corresponds to a band, which in turn determines the final score for the indicator. Using a 3-2-1 point scale for total ban, partial statutory restriction and voluntary agreement/self-regulation, respectively, there is a maximum number of 30 points for each beverage type (3 points x 10 advertising platforms), or a total of 90 points for beer, wine and spirits combined. Bands are then created (for example, band 0: 0 points, band 1: 1–22 points, band 2: 23–44 points, band 3: 45–67 points, band 4: 68–90 points) and points assigned to each band.

6a.1 Legally binding restrictions on alcohol advertising

☐ Band 4 (68–90 points) (4 p.)	☐ Band 3 (45–67 points) (3 p.)	☐ Band 2 (23–44 points) (2 p.)	☐ Band 1 (1–22 points) (1 p.)	☐ Band 0 (0 points) (0 p.)

Multiplier x 3

6a.2 Legally binding restrictions on product placement

☐ Band 4 (21–27 points) (4 p.)	☐ Band 3 (14–20 points) (3 p.)	☐ Band 2 (7–13 points) (2 p.)	☐ Band 1 (1–6 points) (1 p.)	☐ Band 0 (0 points) (0 p.)

Multiplier x 3

6a.3 Legally binding restrictions on industry sponsorship for sporting and youth events

☐ Band 4 (14–18 points) (4 p.)	☐ Band 3 (9–13 points) (3 p.)	☐ Band 2 (5–8 points) (2 p.)	☐ Band 1 (1–4 points) (1 p.)	☐ Band 0 (0 points) (0 p.)

Multiplier x 3

6a.4 Legally binding restrictions on sales promotions by producers, retailers and owners of pubs and bars

☐ Band 4 (21–27 points) (4 p.)	☐ Band 3 (14–20 points) (3 p.)	☐ Band 2 (7–13 points) (2 p.)	☐ Band 1 (1–6 points) (1 p.)	☐ Band 0 (0 points) (0 p.)

Multiplier x 3

7.	**Pricing policies (*see* also rubric 7a) (maximum 70 p.)**		
**7.1**	**Adjustment of taxation level for inflation**<br>*This is to indicate whether the level of taxation (excise tax or special tax on alcohol other than excise tax) for alcoholic beverages is adjusted for inflation.*		
	☐ At least two types of beverage (4 p.)	☐ One type of beverage (beer, wine or spirits) (2 p.)	☐ No (0 p.)
	Multiplier x 3		
**7.2**	**Affordability of alcoholic beverages see subric 7a**		
	Multiplier see rubric 7a		
**7.3**	**Other price measures**<br>*This is to indicate whether there are any price measures other than taxation in a given country. Price measures other than taxation mean, for example, regulation of the price of non-alcoholic and alcoholic beverages, such as making a non-alcoholic beverage cheaper than an alcoholic beverage. They include: minimum price policy, additional levy on specific products (such as alcopops), requirement to offer non-alcoholic beverages at a lower price, ban on below-cost selling, or ban on volume discounts.*		
	Minimum price policy	☐ Yes (3 p.)	☐ No (0 p.)
	Additional levy on specific products	☐ Yes (3 p.)	☐ No (0 p.)
	Requirement to offer a non-alcoholic beverage at a lower price than an alcoholic beverage on the premises	☐ Yes (2 p.)	☐ No (0 p.)
	Ban on below-cost selling	☐ Yes (3 p.)	☐ No (0 p.)
	Ban on volume discounts	☐ Yes (3 p.)	☐ No (0 p.)
	Multiplier x 3		

7a.	**Pricing policies**				
**7a.1**	**Affordability of alcoholic beverages**<br>*A band is ascertained according to the price indices of different types of beverage.*<br>*The price index is a modification of the affordability measure first introduced by Brand et al. (2007), and is defined as follows:*<br>*Price index=10 000 × (Price (calculated based on standard containers of 50 cl beer,75 cl wine and 70 cl spirits)(€))/(Gross national income at PPP per capita (current international $))*<br>*The price index is calculated separately for beer, wine and spirits, and an overall score for the affordability indicator is determined using the banding approach.*				
	☐ Band 4 (13–16 points) (4 p.)	☐ Band 3 (10–12 points) (3 p.)	☐ Band 2 (7–9 points) (2 p.)	☐ Band 1 (4–6 points) (1 p.)	☐ Band 0 (≤ 3 points) (0 p.)
	Multiplier x 4				

8.	**Reducing the negative consequences of drinking and alcohol intoxication (maximum 16 p.)**	
**8.1**	**Server training**<br>*Server training is provided on a regular basis to bar staff and staff at special events to give them skills and knowledge about alcohol harm and safe serving practices.*	
	☐ Yes (3 p.)	☐ No (0 p.)
	Multiplier x 2	

**8.2**	**Health warning labels**<br>*Health warning labels are present with information on the dangers associated with the use of the product.*		
	Health warning labels are legally required on alcohol advertisements	☐ Yes (2 p.)	☐ No (0 p.)
	Health warning labels are legally required on containers/bottles of alcoholic beverages	☐ Yes (3 p.)	☐ No (0 p.)
	Multiplier x 2		
**9.**	**Reducing the public health impact of illicit alcohol and informally produced alcohol (maximum 30 p.)**		
**9.1**	**Use of duty paid or excise stamps on alcohol containers**<br>*Excise stamps on alcohol containers are used by national customs to signify that the excise tax has been paid.*		
		☐ Yes (3 p.)	☐ No (0 p.)
	Multiplier x 3		
**9.2**	**Estimates of unrecorded alcohol consumption**<br>*Unrecorded alcohol is alcohol that is not taxed and is outside the usual system of governmental control, such as home- or informally produced alcohol (legal or illegal), smuggled alcohol, surrogate alcohol (alcohol not intended for human consumption), or alcohol obtained through cross-border shopping which is recorded in a different jurisdiction.*<br><br>*Regular estimates of the consumption of unrecorded alcohol may be available in a country based on expert opinion, research focused on unrecorded alcohol consumption, indirect estimates using government data on confiscated/seized alcohol, indirect estimates using survey data or indirect estimates using other data.*		
		☐ Yes (3 p.)	☐ No (0 p.)
	Multiplier x 3		
**9.3**	**Legislation to prevent illegal production and sale of alcoholic beverages**<br>*National legislation is in place to prevent the illegal production and/or sale of home- or informally produced alcoholic beverages.*		
	Legislation exists to prevent illegal production of alcoholic beverages	☐ Yes (3 p.)	☐ No (0 p.)
	Legislation exists to prevent illegal sale of alcoholic beverages	☐ Yes (3 p.)	☐ No (0 p.)
	Multiplier x 2		
**10.**	**Monitoring and surveillance (maximum 90 p.)**		
**10.1**	**National monitoring system**<br>*This is to indicate whether there is a national system for monitoring alcohol-related harm.*		
	National system for monitoring includes data on:	☐ alcohol consumption (including regular national surveys of consumers and abstainers in the general population) (3 p.)<br>☐ health consequences (3 p.)<br>☐ social consequences (3 p.)<br>☐ alcohol policy responses (3 p.)<br>☐ sales data (3 p.)	☐ No national monitoring system (0 p.)

	Regular reports are published using data from national monitoring system	☐ Yes (4 p.)	☐ No (0 p.)
	An institution/organization/department has the mandated function of a national monitoring centre or a person has the mandated function of monitoring the situation on alcohol and health	☐ Yes (4 p.)	☐ No (0 p.)
	Multiplier x 3		
**10.2**	**National surveys**<br>*This is to indicate whether there are national surveys of the rates of heavy episodic drinking (binge drinking) among adults and of alcohol consumption among young people (including school-based surveys).*		
	Surveys of heavy episodic drinking are carried out	☐ Yes (4 p.)	☐ No (0 p.)
	Surveys of young adult and underage drinkers are carried out	☐ Yes (3 p.)	☐ No (0 p.)
	Multiplier x 3		

The WHO Regional Office for Europe

The World Health Organization (WHO) is a specialized agency of the United Nations created in 1948 with the primary responsibility for international health matters and public health. The WHO Regional Office for Europe is one of six regional offices throughout the world, each with its own programme geared to the particular health conditions of the countries it serves.

Member States

Albania
Andorra
Armenia
Austria
Azerbaijan
Belarus
Belgium
Bosnia and Herzegovina
Bulgaria
Croatia
Cyprus
Czechia
Denmark
Estonia
Finland
France
Georgia
Germany
Greece
Hungary
Iceland
Ireland
Israel
Italy
Kazakhstan
Kyrgyzstan
Latvia
Lithuania
Luxembourg
Malta
Monaco
Montenegro
Netherlands
Norway
Poland
Portugal
Republic of Moldova
Romania
Russian Federation
San Marino
Serbia
Slovakia
Slovenia
Spain
Sweden
Switzerland
Tajikistan
The former Yugoslav Republic of Macedonia
Turkey
Turkmenistan
Ukraine
United Kingdom
Uzbekistan

Policy in action

World Health Organization Regional Office for Europe
UN City, Marmorvej 51, DK-2100 Copenhagen Ø, Denmark
Tel.: +45 45 33 70 00 Fax: +45 45 33 70 01
E-mail: euwhocontact@who.int Website: www.euro.who.int
